# *Elongation Factor 1 alpha1* and Genes Associated with Usher Syndromes Are Downstream Targets of GBX2

**DOI:** 10.1371/journal.pone.0047366

**Published:** 2012-11-08

**Authors:** David A. Roeseler, Shrikesh Sachdev, Desire M. Buckley, Trupti Joshi, Doris K. Wu, Dong Xu, Mark Hannink, Samuel T. Waters

**Affiliations:** 1 Division of Biological Sciences, University of Missouri, Columbia, Missouri, United States of America; 2 Christopher S. Bond Life Sciences Center, University of Missouri, Columbia, Missouri, United States of America; 3 Department of Biochemistry, University of Missouri, Columbia, Missouri, United States of America; 4 Department of Computer Science, University of Missouri, Columbia, Missouri, United States of America; 5 Informatics Institute, University of Missouri, Columbia, Missouri, United States of America; 6 Laboratory of Molecular Biology, NIDCD, National Institutes of Health, Bethesda, Maryland, United States of America; Wayne State University, United States of America

## Abstract

*Gbx2* encodes a DNA-binding transcription factor that plays pivotal roles during embryogenesis. Gain-and loss-of-function studies in several vertebrate species have demonstrated a requirement for *Gbx2* in development of the anterior hindbrain, spinal cord, inner ear, heart, and neural crest cells. However, the target genes through which GBX2 exerts its effects remain obscure. Using chromatin immunoprecipitation coupled with direct sequencing (ChIP-Seq) analysis in a human prostate cancer cell line, we identified cis-regulatory elements bound by GBX2 to provide insight into its direct downstream targets. The analysis revealed more than 286 highly significant candidate target genes, falling into various functional groups, of which 51% are expressed in the nervous system. Several of the top candidate genes include *EEF1A1*, *ROBO1*, *PLXNA4*, *SLIT3*, *NRP1*, and *NOTCH2*, as well as genes associated with the Usher syndrome, *PCDH15* and *USH2A*, and are plausible candidates contributing to the developmental defects in *Gbx2^−/−^* mice. We show through gel shift analyses that sequences within the promoter or introns of *EEF1A1*, *ROBO1*, *PCDH15*, *USH2A* and *NOTCH2*, are directly bound by GBX2. Consistent with these in vitro results, analyses of *Gbx2^−/−^* embryos indicate that *Gbx2* function is required for migration of *Robo1*-expressing neural crest cells out of the hindbrain. Furthermore, we show that GBX2 activates transcriptional activity through the promoter of *EEF1A1*, suggesting that GBX2 could also regulate gene expression indirectly via EEF1A. Taken together, our studies show that GBX2 plays a dynamic role in development and diseases.

## Introduction

Transcriptional regulation of gene expression in cells during development is critical in defining their positional identity and allowing their differentiation into various cell types, tissues and organs. *Gbx1* and *Gbx2* encode the two members of the Gbx family of DNA-binding transcription factors that are expressed during embryogenesis in many vertebrate and non-vertebrate species [1]–[3]. While few studies have provided knowledge about *Gbx1* during development [3]–[6], misexpression and loss-of-function studies have revealed distinct spatiotemporal requirements for *Gbx2* expression [7]–[10]. *Gbx2* RNA is broadly expressed in all three germ layers throughout the posterior region of the embryo during gastrulation, with an anterior expression border marking the prospective midbrain-hindbrain boundary (MHB) [7]. Patterning and growth of the midbrain and anterior hindbrain (cerebellum) is regulated in part by the secreted signaling molecule fibroblast growth factor (Fgf) 8, originating from a distinct anatomical constriction positioned at the interface between the prospective midbrain and hindbrain known as the isthmic organizer [11]–[13]. Studies in several vertebrate species have demonstrated a requirement of *Gbx2* for the induction of genes expressed at the MHB as well as in determining the position of the isthmic organizer and the induction of genes expressed at the MHB; for reviews see [14], [15].

More recent studies have revealed the importance of *Gbx2* in patterning the anteroposterior (AP) axis of the hindbrain [10], [16], [17]. During the early stages of neurulation, the hindbrain region is transiently segmented into eight morphologically distinct compartments known as rhombomeres, from which distinct brain structures and neuronal populations are derived [18], [19]. For example, rhombomere (r) 1 gives rise to the cerebellum, whereas r2 and r3 give rise to motor neuron cell bodies of cranial nerve V. Between embryonic day (E) 8.5 and E9.5, *Gbx2* is primarily expressed in the anterior hindbrain and caudal neural tube [20], [21]. In *Gbx2* nulls, r1–r3 are not defined and they die postnatally due to forebrain and cerebellar defects [7]. In *Gbx2^neo^* hypomorphic embryos, in which *Gbx2* level is decreased to 6–10% of normal, r1 and r2 development is abnormal and their derivatives the cerebellum and cranial nerve V, respectively, are consistently malformed [16].

**Figure 1:**
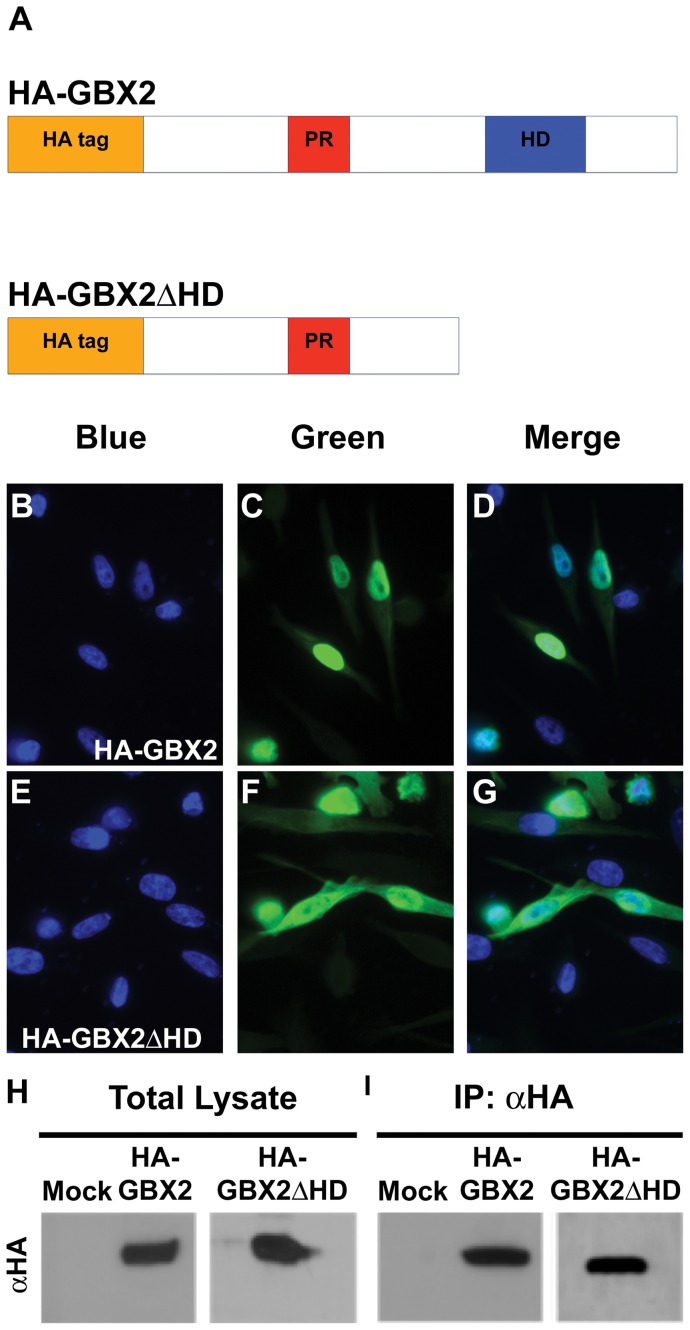
GBX2 overexpression and ChIP in human PC-3 cells. (A) Schematic representation of the HA-GBX2 and HA-GBX2ΔHD recombinant proteins containing the proline-rich region (PR), DNA-binding homeodomain (HD), and the HA epitope tag located at the amino terminus. Immunoflouresence of transiently transfected human PC-3 cells with *HA-GBX2* (C, D), and, *HA-Gbx2*Δ*HD* (F, G). Blue channel identifies DAPI staining in the nucleus (B, E). Green channel identifies GFP-GBX2 fusion proteins. (D, G) Merge displays nuclear localization of GFP-GBX2 fusion proteins. Western blots of total lysates (H) and HA-immunoprecipitated samples (I) from mock, *HA-Gbx2*, and *HA-Gbx2*Δ*HD* transfected PC-3 cells.

The expression of *Gbx2* in the hindbrain during segmentation is similar among vertebrate species, and loss-of-function studies in mouse and zebrafish have demonstrated evolutionarily conserved functions for *Gbx2* in A-P patterning within the anterior hindbrain. While the apparent function of *Gbx2* at the MHB is not conserved between mouse and zebrafish, recent studies using a *gbx2* antisense morpholino in zebrafish have shown that the anterior hindbrain is significantly truncated between the anterior boundaries of r1 and r3, and cranial nerve V motor neuron cell bodies in r2 and r3 are severely disorganized [5], [10]. Furthermore, regulation of motor neuron development by *Gbx2* is not limited to the anterior hindbrain. Recent fate mapping studies in the caudal neural tube of mouse embryos show that both ventral motor neurons and interneurons are derived from the *Gbx2* lineage and spinal cord patterning is globally affected by the loss of *Gbx2* [8].

**Figure 2:**
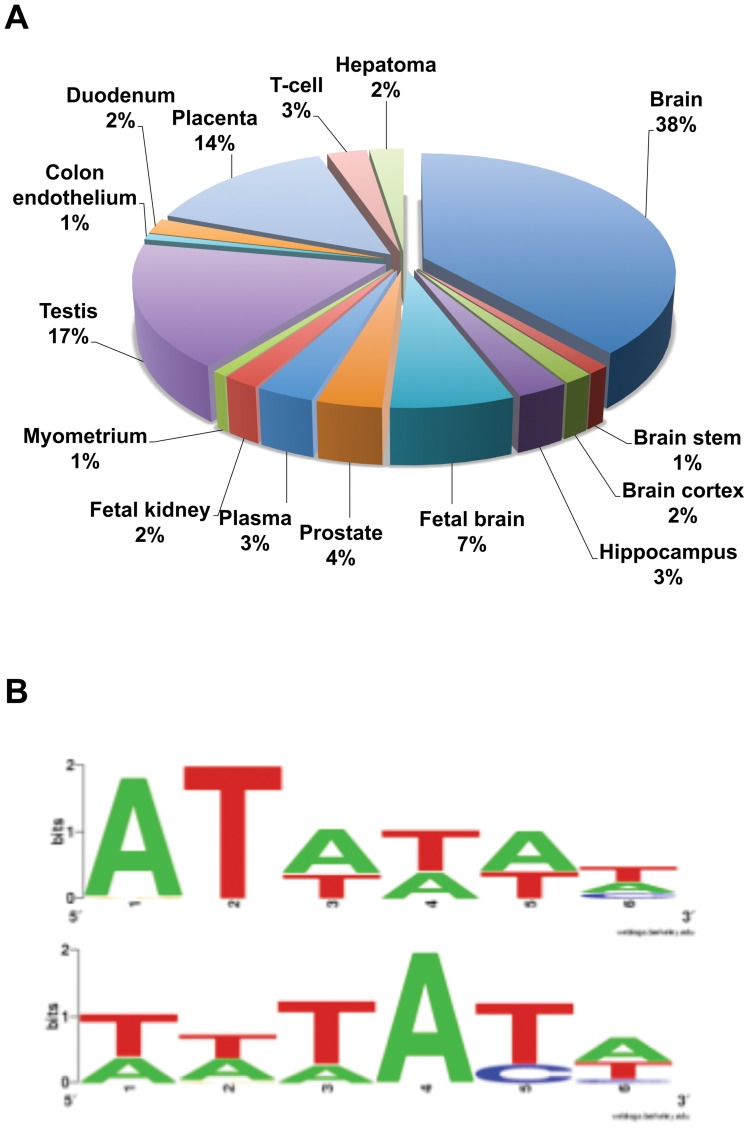
Bioinformatic analysis of the top 286 GBX2 target genes. (A) Tissue expression analysis for the top 286 genes targeted by GBX2 determined by DAVID. Of the top 286 genes targeted by GBX2, 51% are expressed in the nervous system: 38% brain, 1% brain stem, 2% brain cortex, 3% hippocampus, 7% fetal brain. (B) The top two GBX2 DNA-binding consensus motifs bioinformatically determined by Motif Sampler analysis of GBX2 ChIP-Seq target sequence fragments.

**Table 1:** ChIP-Seq identified GBX2 targets.

Candidate Gene ID	P value	Fold enrichment	Number of tags	Consensus motif	Entrez ID
**NFE2L2**	**2.6×10^−270^**	**26.18**	**376**	**yes**	**4780**
EEF1A1	1.2×10^−36^	6.26	154	yes	1915
OTUD7B	4.1×10^−13^	90.17	8	yes	56957
ULK4	1.2×10^−12^	80.17	8	yes	54986
OR5H1	3.0×10^−10^	12.72	19	yes	26341
ACSL1	2.9×10^−9^	45.09	6	no	2180
ELMO1	2.4×10^−9^	42.87	7	no	9844
SOCS4	9.7×10^−8^	45.09	5	yes	122809
PPP3CB	8.9×10^−8^	18.52	9	yes	5532
**LAMA2**	**6.2×10^−8^**	**51.67**	**5**	**no**	**3908**
**PLXNA4**	**4.3×10^−8^**	**33.07**	**6**	**no**	**91584**
**ROBO1**	**3.7×10^−8^**	**56.36**	**5**	**yes**	**6091**
SCFD2	3.7×10^−8^	56.36	5	yes	152579
TNFRSF21	3.7×10^−8^	56.36	5	no	27242
ERC2	3.7×10^−8^	56.36	5	yes	26059
TGFA	3.7×10^−8^	45.09	5	yes	7039
GUSBL1	3.5×10^−8^	39.68	6	yes	387036
VCL	2.1×10^−8^	24.8	7	yes	7414
**SYT2**	**1.7×10^−8^**	**27.56**	**7**	**no**	**127833**
**RTN1**	**9.2×10^−7^**	**15.34**	**8**	**yes**	**6252**
**PARD3**	**9.0×10^−7^**	**12.02**	**10**	**yes**	**56288**
**FGF12**	**8.2×10^−7^**	**13.23**	**9**	**yes**	**2257**
**NRP1**	**5.3×10^−7^**	**15.03**	**7**	**yes**	**8829**
**BRE**	**1.9×10^−7^**	**45.09**	**5**	**yes**	**9577**
**SBF2**	**1.8×10^−7^**	**45.09**	**5**	**no**	**81846**
IL1RAP	1.2×10^−7^	45.09	5	no	3556
MYST4	1.1×10^−7^	22.05	7	yes	23522
**NOTCH2**	**9.1×10^−6^**	**14.7**	**6**	**yes**	**4853**
**PCDH15**	**7.3×10^−6^**	**19.84**	**5**	**yes**	**65217**
**GRIA1**	**5.3×10^−6^**	**8.27**	**9**	**yes**	**2890**
**USH2A**	**3.6×10^−6^**	**13.78**	**6**	**yes**	**7399**
**KCNB2**	**2.5×10^−6^**	**15.31**	**7**	**yes**	**9312**
**SLIT3**	**1.4×10^−6^**	**21.2**	**6**	**no**	**6586**
TBC1D5	1.2×10^−6^	2.98	82	yes	9779

Genes targeted by GBX2 in human PC-3 cells based on ChIP-Seq fragments aligned to the hg18 build of the human genome on the UCSC Human Genome Browser. Bold text indicates GBX2 targets expressed in the nervous system.

In addition to the neural tube, *Gbx2* is expressed in the otic placode, which develops into the inner ear [22], [23]. The inner ear is a complex sensory end organ for detecting sound, head position and movements. Highly differentiated mechanosensory hair cells within various sensory patches of the inner ear are critical for the transduction of mechanical forces arising from sound waves and movements [24]. Perturbation of normal organization and growth of hair bundles within the inner ear is a feature of many congenital diseases such as Usher syndrome, in which affected individuals have sensory defects such as blindness, hearing loss and vestibular deficits [25]–[28]. Loss-of-function and misexpression studies have shown that *Gbx2* is essential for development of the inner ear sensory organs [29], [30]. Moreover, the highly variable and incompletely penetrant inner ear malformations seen in *Gbx2^−/−^* embryos have close parallels with those seen in hearing-impaired patients [30]–[33].

**Figure 3:**
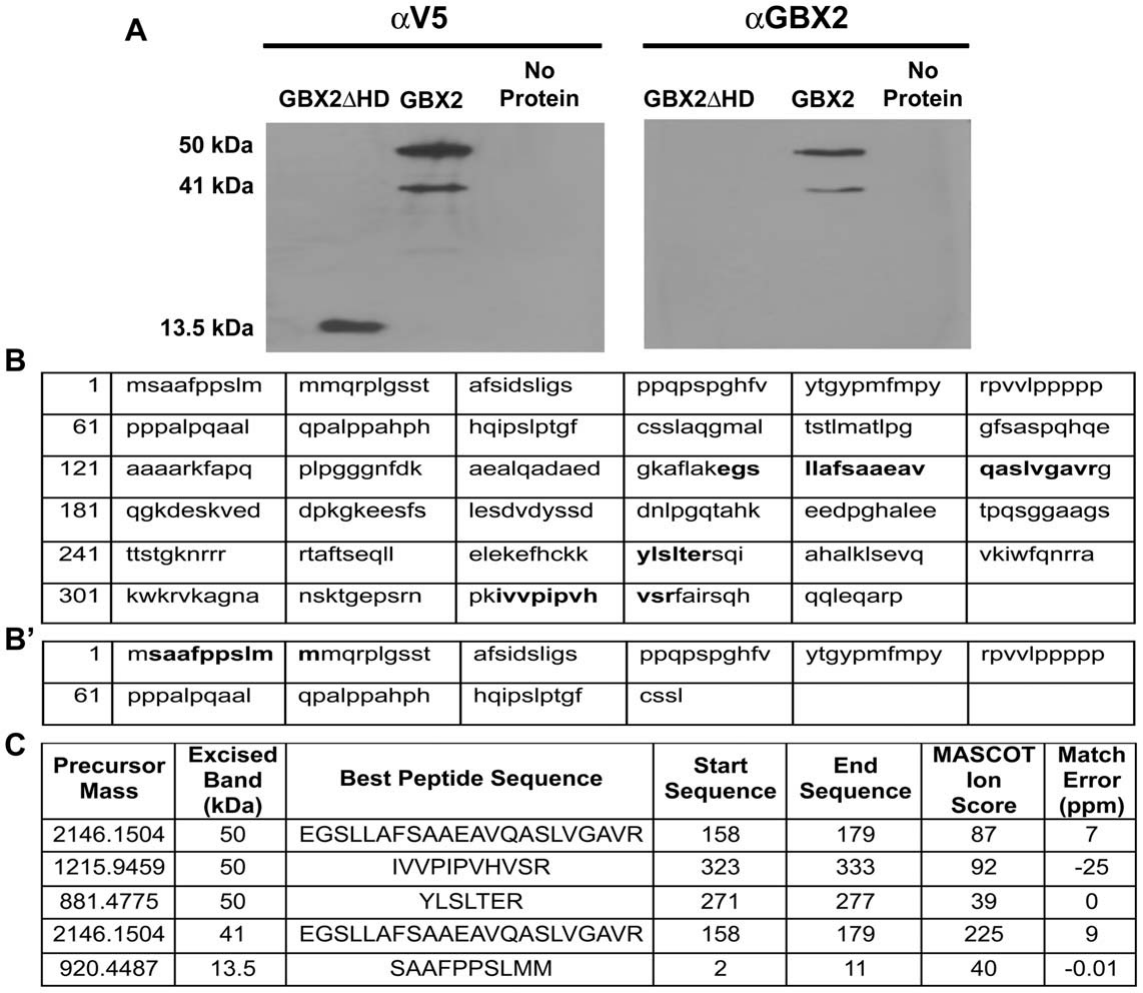
Confirmation of murine GBX2 by Western blot and mass spectral analysis. (A) Western analysis of recombinant GBX2 proteins. The amino acid sequences for GBX2 (B) and GBX2Δ*HD* (B') recombinant proteins. Bold type indicates matched peptides identified by mass spectrometry. (C) MS/MS fragmentation data table includes: precursor mass (peptide chosen for MS/MS), approximate weight of the band analyzed by mass spectrometry, peptide sequence, location of the peptide in the protein sequence, the Mascot ion score, and the mass error for each peptide sequence. The low mass error score in parts per million (ppm = {[observed mass – theoretical mass]/theoretical mass}×10^6^) suggests that the observed mass matches the theoretical mass.

Several recent studies have demonstrated essential roles for *Gbx2* in the induction, migration and patterning of cranial as well as cardiac neural crest (NC) cells [34], [35]. Importantly, loss of *Gbx2* function modulates the Slit/Robo signaling pathway, resulting in abnormal NC cell migration and abnormalities resembling congenital diseases such as DiGeorge syndrome, in which the main cause of death is congenital heart defects. The diverse spatiotemporal functions of *Gbx2* rely on its ability to interact with promoters and enhancers of cognate target genes. Therefore, identifying direct targets of Gbx2 is important for understanding the molecular mechanisms and signaling pathways that contribute to the striking developmental deficits caused by the absence of this gene [36], [37].

**Figure 4:**
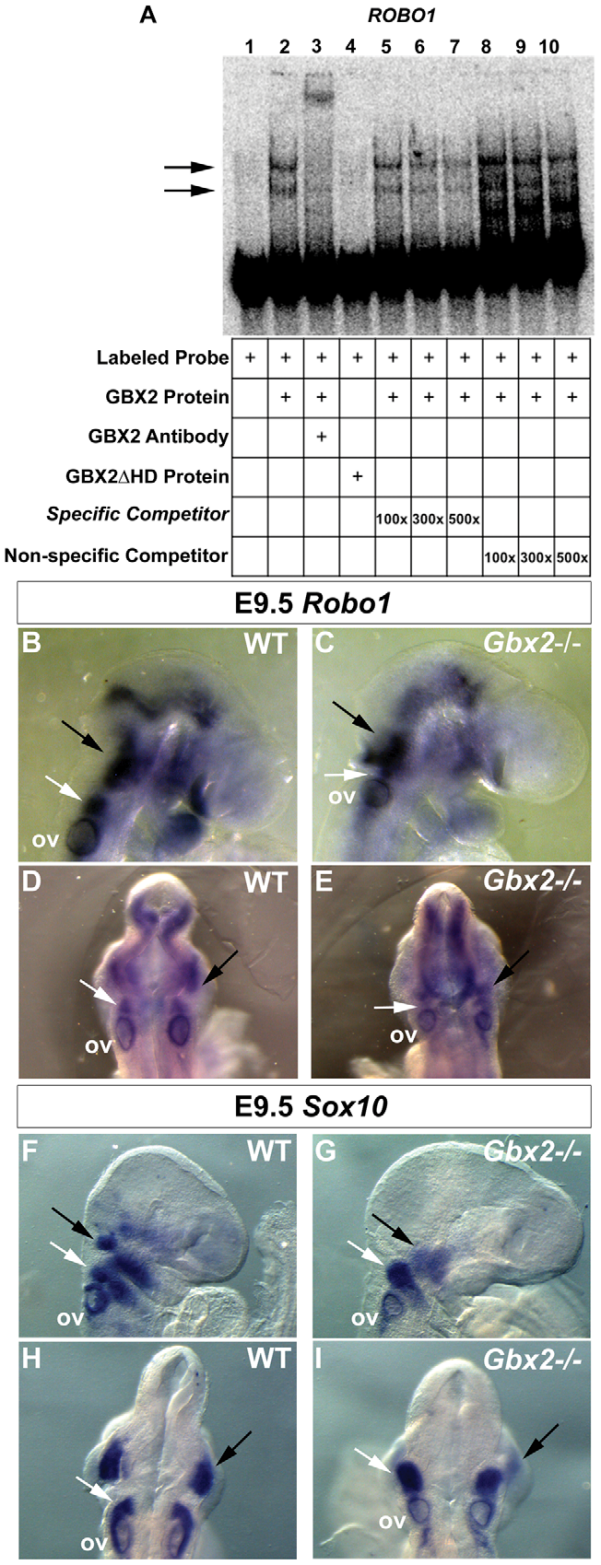
Binding to *ROBO1* and regulation of NC cell patterning by GBX2. (A) Gel-shift analysis for identified GBX2 target *ROBO1*. A reduction in the mobility of [ÿ -32P] ATP labeled *ROBO1* 100-mer probe is observed with the addition of GBX2 (black arrows), whereas no shift is observed with the addition of GBX2ΔHD (compare lane 2 to lane 4). A supershift is observed in lane 3 with the addition of anti-GBX2. Addition of identical *ROBO1* 100-mer unlabeled specific competitor probe at 100x, 300x, and 500x molar concentrations in lanes 5–7. Addition of *ROBO1* 45-mer unlabeled non-specific competitor probe, omitting the GBX2 DNA-binding sequence in lanes 8–10. (B–I) Whole mount in situ hybridization for *Robo1* (B–E) and the migrating neural crest marker, *Sox10* (F–I) at gestational stage E9.5, demonstrates abnormal expression in *Gbx2^−/−^* mutants. Image analysis of embryos in a right lateral view (B,C) and dorsal view (D,E) reveals a reduction in expression of *Robo1* in rhombomere 4 (compare white arrows in B and D to C and E) and disorganized expression in the rhombomere 1 domain (compare black arrows in B and D to C and E) in *Gbx2* ^–/–^ mutants compared to the WT control. Lateral and dorsal views reveal a reduction in *Sox10* expression within the otic vesicle (compare F and H to G and I). Two distinct streams of NCCs into pharyngeal arch 1 and pharyngeal arch 2 are defined within control embryos (F, H) whereas in *Gbx2* ^−/−^ mutants (G,I) the NC streams appear disrupted. In the mutant, expression of *Sox10* within the NC stream into presumptive pharyngeal arch 1 is significantly downregulated (compare black arrows in F and H to G and I), and the NCCs within the pharyngeal arch 2 appear to be compacted more posteriorly, truncating the stream (compare white arrows in F and H to G and I) when compared to the WT control. ov  =  otic vesicle.

In this report, we have identified several GBX2 candidate target genes including *ROBO1*, *PLXNA4*, *SLIT3*, *NRP1*, *PCDH15*, *USH2A*, *NOTCH2*, *and EEF1A1* that are likely to contribute many of the developmental defects observed in *Gbx2* mutant organisms. Identification of these direct target genes should advance our understanding of the functions of GBX2 and provide greater insight into the relationships of GBX2 and various congenital diseases such as Usher and DiGeorge Syndromes.

## Results

### Identification of GBX2 candidate target genes

Sequences of the Gbx family of homeobox genes are highly conserved between species and are generally more than 75% related at the amino acid level within a specific class. Importantly, mouse and human GBX2 share 98% overall sequence identity and 100% identity in the 60 amino acids of the DNA-binding homeodomain [2], [38]. To identify candidate target genes directly regulated by GBX2, we preformed chromatin immunoprecipitation assays coupled with high-throughput sequencing (ChIP-Seq), using PC-3 cells, a human prostate cancer line. This cell line was selected because previous studies have shown that the expression of *GBX2* is consistently upregulated in PC-3 cells when compared to normal prostate [39]. In addition, PC-3 cells have been used to identify a known binding target of GBX2, *IL-6* [37]. A critical aspect of ChIP is the ability of the antibody to specifically immunoprecipitate the protein of interest. To generate a form of GBX2 recognizable by an antibody with great specificity, we generated two cDNA constructs containing the well characterized, and highly immunoreactive N-terminal hemagglutinin (HA) epitope tag. Both full-length mouse *Gbx2* and a truncated *Gbx2* lacking the c-terminal region encoding the DNA-binding homeodomain (amino acids 221–348), *HA-Gbx2* and *HA-Gbx2*Δ*HD*, respectively, were subcloned into the pCMV-HA mammalian expression vector (Fig. 1A). The expression vectors were transfected into PC-3 cells (ATCC, CRL-1435), which were then examined for nuclear expression of each fusion protein by immunoflourescence. Both proteins were primarily localized to the nucleus (Fig. 1C, F). Nuclear localization was verified by DAPI staining (Fig. 1B, E, D, G).

To determine which cis-regulatory elements are bound by HA-GBX2, protein-DNA complexes were immunoprecipitated from PC-3 cells expressing HA-GBX2 with a HA-specific antibody. Anti-HA immunoprecipitations from HA-GBX2ΔHD transfected cells were treated in parallel (Fig. 1. H, I). Purified ChIP DNA and total input DNA were sequenced using the high-throughput Illumina sequencing platform. We obtained 18 million reads for each sample as well as for the control input DNA sample. Sequences were aligned to the hg18 build of the human genome using the UCSC Human Genome Browser [40]. Fragment density maps were generated using the Model-based Analysis of ChIP-Seq data (MACS) software identifying the HA-GBX2-enriched DNA fragments over HA-GBX2ΔHD-enriched DNA fragments and total input controls (data not shown) [41]. Using the MACS software and only considering sequences (tags) located within the candidate gene 1kb upstream of the initiation site or downstream of the termination site, we identified greater than 1,000 genes bound by GBX2. Interestingly, many of the GBX2-bound sequences were localized within introns of the genes. Additional selection parameters from data generated by MACS, such as the number of sequence tags (≥5), fold enrichment over background, and a *p*-value of ≤1×10^−5^, were used to reduce the number of candidate target genes to 286 (listed in Table S1). Thirty-four of the top candidate targets identified based on our stringent selection parameters are reported in Table 1. Importantly, many of the candidate genes could be involved in the molecular mechanisms and signaling pathways underlying the defects associated with deficient *Gbx2* expression during embryonic development. The genes were grouped together based on the tissues in which they are expressed using DAVID (Fig. 2A, [42]). Our analysis showed that the candidate genes are expressed in many different tissues, including placental (14%), reproductive organs such as prostate or testis (20.6%), and neural (51%) tissues. The large percentage of genes found that are expressed in the neural tissue is consistent with the known role of *Gbx2* in numerous mammalian neural-developmental processes [7], [16], [34], [35].

The GBX DNA-binding homeodomain is highly conserved and has been well studied [3], [4]. We analyzed the ChIP-identified sequences to determine if they contained a consensus motif(s) that could represent a novel GBX2 binding site. Several studies have shown that the core sequences of homeodomain binding sites are (AT) rich [43], [44]. Importantly, results from previous studies using mutated oligonucloetides suggest use of an ATTA core sequence by GBX2 [36]. We analyzed the enriched GBX2-bound sequence fragments by Motif Sampler [42], [45] and identified two overrepresented (AT) rich consensus motifs (Fig. 2B). Importantly, the GBX2 binding motifs are consistent with those of other known homeodomain transcription factors [36], [37], [46] and therefore suggest that we have identified two novel binding motifs for GBX2.

### Gel mobility shift assays validate the specificity of the ChIP-Seq identified genes

We next sought to confirm a direct interaction between GBX2 and our identified target fragments. To test whether there is a direct binding interaction between GBX2 and our target gene sequences, we first generated recombinant full-length GBX2 and truncated (GBX2ΔHD amino acids 1–94) proteins in *E. coli* then assessed their presence by Western blotting using an epitope-specific anti-V5 and an anti-GBX2 antibody. We detected bands at the expected sizes of 41 kDa and 13.5 kDa (Fig. 3A) using the epitope-specific anti-V5 antibody. However, since the epitope used to generate the anti-GBX2 antibody (amino acids 181–193) is located 3′ of the sequence used to generate GBX2ΔHD, only the full-length protein is detected (Fig. 3A). In addition to the 41 kDa band, a band at approximately 50 kDa was also consistently observed by both antibodies. To verify the proteins in our Western blots, mass spectral analyses were performed in order to confirm both full-length and truncated bands. Tandem MS/MS mass spectral analyses of peptide fragments excised from the 41 kDa, 50 kDa and 13.5 kDa gel bands indicated amino acid sequences of EGSLLAFSAAEAVQASLVGAVR for the 41 kDa band (ion score of 225) and the 50 kDa band (ion score of 87; score >36 indicates 95% confidence) and the fragment SAAFPPSLMM (ion score of 40) for the 13.5 kDa band (Fig. 3B, B', C). Thus, the MS/MS ion scores indicate with high confidence that the 41 kDa, 50 kDa, and 13.5 kDa bands are all GBX2.

Next, we examined binding of GBX2 to the ChIP-Seq enriched target sequences in the top thirty-four candidate genes by electrophoretic mobility shift assays (EMSA) using recombinant full-length GBX2 and GBX2ΔHD proteins and radiolabeled oligonucleotide sequences containing the identified GBX2 DNA-binding sites. The target genes were selectively analyzed based on their known involvement with phenotypes associated with *Gbx2* null mutants such as patterning of neural crest cells, development of the anterior hindbrain and proper organogenesis during embryonic development.

### Regulation of Slit/Robo signaling in neural crest cells

*ROBO1* and *SLIT3* were two of our top candidate target genes. However, *ROBO1* ranked higher in significance than *SLIT3* based on our parameters used to select target genes and it is bound by GBX2 through the consensus motif (Table 1). The Slit/Robo signaling pathway has been implicated in neuronal and non-neuronal developmental processes [34], [47]. In mammals, Robo1 expression is complementary to all Slit proteins (1–3) and their interaction mediates repulsive guidance cues during neuronal and axonal migration [48]. Although all mammalian Slit proteins are widely expressed in the central nervous system, recent genetic studies have shown that *Slit3* is dispensable in neural development [49]. As such, *Slit3* is required for non-neural developmental processes such as genesis of the diaphragm, kidneys and blood vessels [34]. Therefore, since SLIT3 mediates signals through ROBO1 receptors, and *ROBO1* has a higher level of significance as a candidate target gene of GBX2, we first examined the ability of GBX2 to directly bind and regulate *ROBO1*.

We preformed an EMSA analysis on labeled *ROBO1* oligonucleotide target sequence. Consistent with our data above (Fig. 3), we observed two shifted complexes when labeled *ROBO1* oligonucleotide probe was incubated with full-length GBX2 protein (Fig. 4A, lane 2). No changes in mobility were seen when labeled *ROBO1* oligonucleotide was incubated with the control GBX2ΔHD protein (Fig. 4A, lane 4). We confirmed binding of recombinant GBX2 to the labeled oligonucleotide probe by supershift analysis using a GBX2-specific antibody, which resulted in a shift of both complexes (Fig. 4A, lane 3). Notably, the addition of identical unlabeled *ROBO1* oligonucleotide in amounts of 100-fold, 300-fold and 500-fold excess resulted in a progressive reduction in the shifted complexes (Fig. 4A, lanes 5–7), whereas, incubation of GBX2 with excess unlabeled oligonucleotide, in which the region containing the DNA-binding sequence is omitted, results in a loss of competition (Fig. 4A, lanes 8–10). Thus, these data indicate that GBX2 protein binds to the *ROBO1* target sequence (Table S2).

As stated above, *Robo1* expression is required for migrating cranial and trunk NC cells and axonal projections of NC-derived trigeminal sensory neurons (nV). Similarly, loss-of-function studies have implicated *Gbx2* in NC cell migration, pharyngeal arch artery defects and development of the NC-derived sensory neurons forming the proximal ganglion of nV. Therefore, we examined if NC migration is perturbed in *Gbx2^−/−^* embryos. We analyzed wild-type and *Gbx2* ^−/−^ embryos by in situ hybridization at E9.5, a stage when NC cells are migrating into pharyngeal arches 1, 2, 3, 4, and 6 [50]. A comparison of *Robo1* expression with the NC cell marker *Sox10* in wild-type embryos at E9.5, suggests that *Robo1* and *Sox10* are co-expressed in the trigeminal ganglion and NC cells streaming from r4 into branchial arch 2 (Fig. 4B, D, F, H, Figure S1) [51]. Consistent with abnormal development of r1–r3 and NC-derived cells within nV of *Gbx2* ^−/−^ embryos, we observed disorganized expression patterns for *Robo1* and *Sox10* in the region of the trigeminal ganglion in *Gbx2* mutant embryos (Fig. 4C, E, G, I). Notably, *Robo1* expression was reduced in cells corresponding to the r4 stream in *Gbx2* mutants (Fig. 4C and E). The normal expression domain of *Sox10* appeared truncated, with fewer *Sox10*-positive cells present in the second branchial arch (Fig. 4G and I). Taken together, these data indicate that (1) *Robo1* expression is reduced in *Gbx2^−/−^* embryos and (2) reduced *Robo1* expression is consistent with perturbation of NC cell migration into branchial arch 2.

### Analyses of GBX2 inner ear target genes

*Gbx2^−/−^* embryos display multiple inner ear defects, ranging from malformation to a complete loss of vestibular and cochlear inner ear structures [30]. Interestingly, our analysis suggested that several genes critical for inner ear development including, *NOTCH2*, *PCDH15* and *USH2A*, are GBX2 target genes. Recent studies have shown that Notch signaling plays multiple roles during inner ear development including the generation of sensory progenitors and hair cells [52]–[54]. *PCDH15* and *USH2A* encode essential structural components of the hair bundles within the cochlear and vestibular sensory hair cells. However, little data is available describing the expression or molecular mechanisms regulating the expression of *Pcdh15* and *Ush2a* during the earliest stages of stereociliary hair cell bundle formation in inner ear development [55], [56].

**Figure 5:**
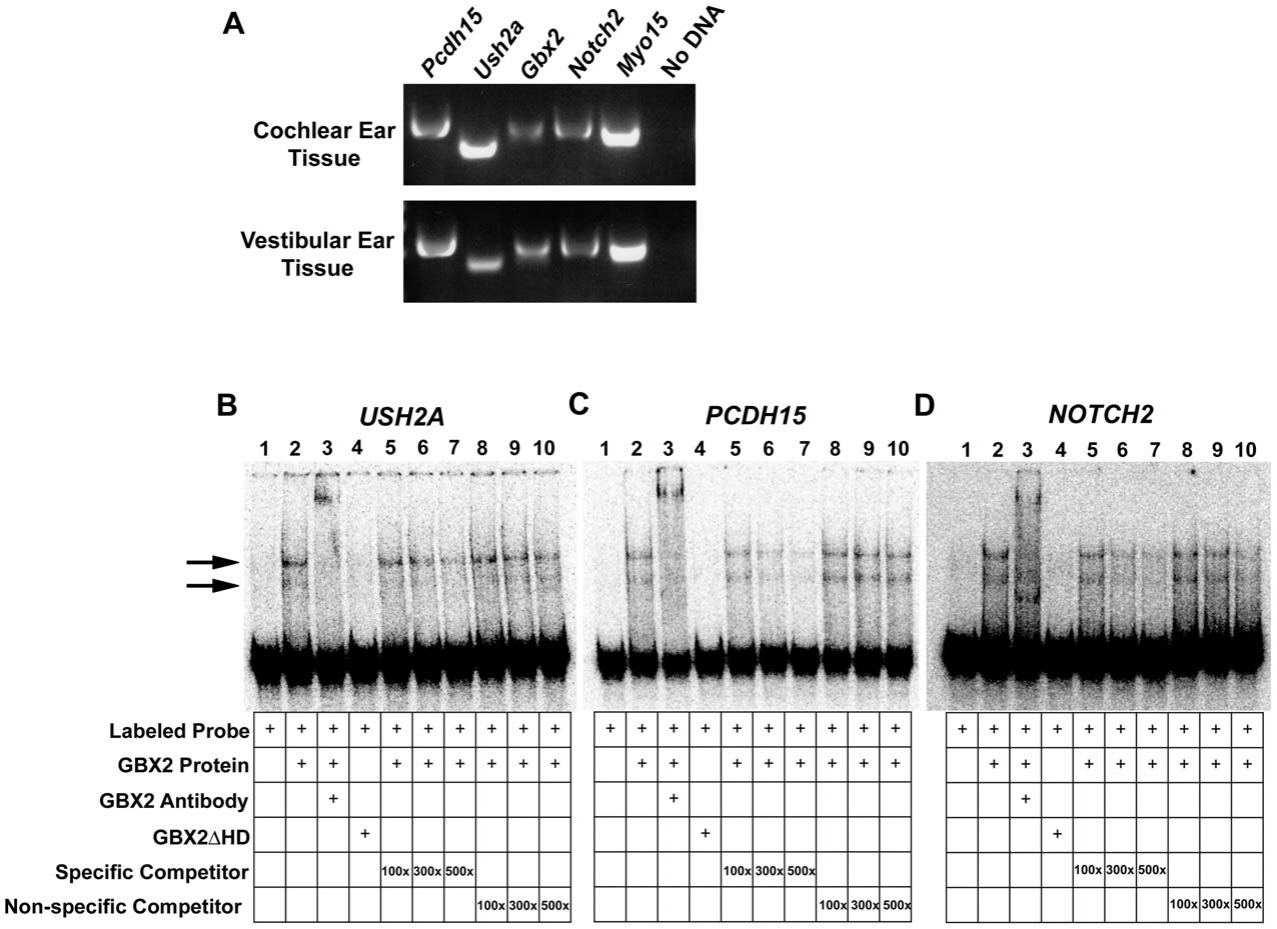
GBX2 directly targets multiple genes associated with Usher syndrome and inner ear development. (A) Reverse transcription (RT)-PCR analysis of *Gbx2* and identified targets, *Pcdh15*, *Ush2a*, and *Notch2*, in E13.5 wild-type mouse cochlear or vestibular inner ear tissues. *Myo15* positive control expression is observed in cochlear and vestibular tissues. (B,C,D) Gel-shift analysis for identified GBX2 targets *USH2A*, *PCDH15*, and *NOTCH2*. A reduction in the mobility of [ÿ -^32^P] ATP labeled *USH2A*, *PCDH15*, and *NOTCH2* 100-mer probes is observed with the addition of GBX2 (black arrows), whereas no shift is observed with the addition of GBX2ΔHD (compare lane 2 to lane 4). A supershift is observed in lane 3 with the addition of anti-GBX2. Addition of identical *USH2A*, *PCDH15*, and *NOTCH2* 100-mer unlabeled specific competitor probes at 100x, 300x, and 500x molar concentrations in lanes 5–7. Addition of *USH2A*, *PCDH15*, and *NOTCH2* 45-mer unlabeled non-specific competitor probe, omitting the GBX2 DNA-binding sequence in lanes 8–10.

We first examined if *Gbx2* is co-expressed with *Pcdh15*, *Ush2a* and *Notch2*, during the earliest stages of hair cell formation in the inner ear of mice at E13.5 using RT-PCR analysis. We extracted RNA from dissected membranous cochlear ducts and vestibular components of wild-type inner ears. *Myo15*, which is localized to cochlear and vestibular hair cells at E13.5, was used as a positive control [57]. Using sequence-specific primers located in two independent exons for each gene, we amplified cDNA products for each target gene, demonstrating that expression of *Gbx2* coincides with that of the identified inner ear targets in both cochlear and vestibular tissues (Fig. 5A). Results from previous expression analysis by *in situ* hybridization at E10.5, suggest that *Gbx2* is primarily expressed in the dorsal-medial region of the otic cup that eventually develops into a non-sensory structure, the endolymphatic duct, which is required for maintaining fluid homeostasis of the inner ear [30]. In contrast, *Gbx2* expression is not detected in other parts of the inner ear by in situ hybridization. While *Gbx2* null inner ears lack the endolymphatic duct, malformations are observed in other sensory and non-sensory components of the inner ear as well. Here, we amplified a cDNA product for *Gbx2* in both cochlear and vestibular tissues at E13.5 suggesting that *Gbx2* is indeed expressed in these tissues and it may have a direct cell autonomous function on sensory hair cell formation.

Next, we determined if the target sequences for *Pcdh15*, *Ush2a* and *Notch2*, are bound by GBX2. Consistent with our bioinformatics analysis (Table 1) and our EMSA data above (Fig. 4), two shifted complexes were formed when labeled oligonucleotide probes for each gene were incubated with full-length GBX2 protein (Fig. 5B–D, lane 2). Similarly, we did not observe a change in mobility when the labeled oligonucleotides were incubated with the control GBX2ΔHD protein (Fig. 5B–D, lane 4), indicating the specificity of GBX2 binding to these genes.

### GBX2 regulates transcriptional activation of *EEF1A1* through direct binding to the core promoter sequence

We identified eukaryotic translation elongation factor 1 alpha 1 (*EEF1A1*) as a candidate target gene of GBX2 (Table 1). eEF1A1 is a GTP-binding protein which has a primary function as an essential house keeping gene by delivering aminoacyl-tRNAs to the ribosome during the elongation step of protein translation [58]. eEF1A1 is broadly expressed and has been shown to be abundant in brain, heart, and skeletal muscle tissue during embryonic development. However, during early postnatal development, eEF1A1 expression is downregulated and replaced by expression of a major isoform of eEF1A, eEF1A2 [59].

**Figure 6:**
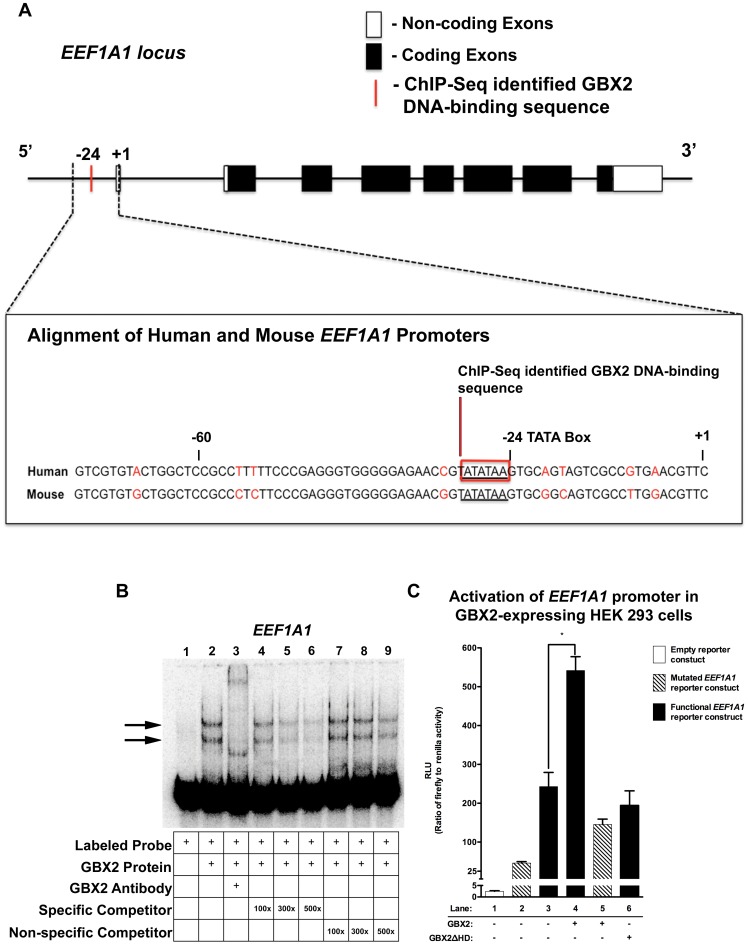
GBX2 binds to and functionally interacts within the *EEF1A1* core promoter. (A) *EEF1A1* locus depicting non-coding exons (white boxes), coding exons (black boxes), and the ChIP-Seq identified location of the GBX2 DNA-binding sequence (red bar). Alignment of the human and mouse ChIP-Seq identified *EEF1A1* promoter region using sequences obtained from Ensembl [61], *EEF1A1* TATA box (underlined sequence) and GBX2 DNA-binding sequence (red box). (B) Gel-shift analysis for identified GBX2 target *EEF1A1*. A reduction in the mobility of [ÿ -^32^P] ATP labeled *EEF1A1* 100-mer probe is observed with the addition of GBX2 (black arrows). A supershift is observed in lane 3 with the addition of anti-GBX2. Addition of identical *EEF1A1* 100-mer unlabeled specific competitor probes at 100x, 300x, and 500x molar concentrations in lanes 4–6. Addition of *EEF1A1* 45-mer unlabeled non-specific competitor probes, omitting the GBX2 DNA-binding sequence in lanes 7–9. (C) *EEF1A1* promoter luciferase reporter assay. HEK 293 cells were transiently transfected with either the empty pGL4.10[*luc2*] vector (white bar), the pGL4.10[*luc2*] vector containing the functional *EEF1A1* promoter sequence and the TATA box (TATATAA; black bars), the pGL4.10[*luc2*] vector containing the mutated *EEF1A1* promoter sequence with a mutated TATA box (TATATAA changed to GCGCGCC; striped bars), and the pGL4.70[*hRluc*] *Renilla* vector. Substantial luciferase activity was observed in cells transfected with the pGL4.10[*luc2*] vector containing the functional *EEF1A1* promoter compared to the empty pGL4.10[*luc2*] reporter construct (compare lane 3 to lane 1). Maximal luciferase activation is observed upon the addition of GBX2 in cells with the pGL4.10[*luc2*] vector containing the functional *EEF1A1* promoter sequence (compare lane 4 to lane 3), and activation is reduced in GBX2Δ*HD* (lane 6) cells and cells expressing GBX2 and the pGL4.10[*luc2*] mutated *EEF1A1* reporter construct (compare lane 4 to lane 5). Luciferase activities were normalized to *Renilla* luciferase activities. * *P* = 0.0047 (two-tailed P value).

The genomic fragment of *EEF1A1* bound by GBX2 was localized to the proximal promoter region (Fig. 6A). Unlike most housekeeping genes, the core promoter of *EEF1A1* contains a putative TATA box sequence (TATATAA) beginning at −24 nucleotides upstream of the transcription initiation site, and has obvious sequence similarity with the consensus GBX2-binding motif (Fig. 2) [60]. The *EEF1A1* core promoter region (−60 to +1) is highly conserved (86.7%) between human and mouse [61] suggesting that regulation of *EEF1A1* by GBX2 may also be conserved across various vertebrate species. Surprisingly, the GBX2-binding site identified by MACS (ATATAA) (Table S2), exhibits significant overlap with the putative TATA box within the *EEF1A1* core promoter.

We examined the binding of GBX2 to the *EEF1A1* core promoter by EMSA. We observed a shift in mobility and a supershift when labeled *EEF1A1* promoter sequence was incubated with full-length GBX2 alone and full-length GBX2 plus anti-GBX2 antibody, respectively (Fig. 6B, lane 2, 3). The addition of unlabeled *EEF1A1* oligonucleotide at amounts of 100-fold, 300-fold and 500-fold excess resulted in a progressive reduction in the shifted complexes (Fig. 6B, lanes 4–6). Furthermore, we did not observe any competition at 100-fold and 300-fold amounts of excess unlabeled oligonucleotide in which the region containing the DNA-binding sequence is omitted, and nominal levels at 500-fold, demonstrating that GBX2 binds the EEF1A1 core promoter sequence (Fig. 6B, lanes 7–9).

Since GBX2 binds to the *EEF1A1* core promoter, we next examined the effect of GBX2 on *EEF1A1* transcriptional activity in GBX2-expressing HEK 293FT cells using pGL4.10[*luc2*]-*EEF1A1* reporter constructs. We utilized two reporter constructs containing (1) the functional *EEF1A1* (TATATAA) or (2) a mutated (GCGCGCC) TATA sequence (Table S2). To establish a basal level of transcriptional activation caused by the presence of the putative TATA box alone, we transfected HEK 293FT cells with the functional *EEF1A1* reporter gene and compared the level of transcriptional activation to cells transfected with the empty control or mutated *EEF1A1* reporter constructs. Transfection of the functional *EEF1A1* reporter construct resulted in a marked increase in transcriptional activity compared to controls (Fig. 6C, 1, 2 and 3), suggesting that the putative TATA box and overlapping ChIP-Seq identified GBX2-binding site within the *EEF1A1* core promoter is critical for transcriptional activity. To determine the effect of GBX2 binding to the core promoter sequence of *EEF1A1*, we co-transfected full-length *Gbx2* with the functional reporter gene. HEK 293FT cells co-transfected with full-length *Gbx2* and the mutated reporter gene were used as a control. Our analyses showed that co-transfection with the full-length *Gbx2* expression plasmid significantly increased transcriptional activity of the functional *EEF1A1* reporter gene (P<0.0047) (Fig. 6C, 3 and 4), whereas co-transfection with the mutated reporter gene resulted in a slight decrease in transcriptional activity (Fig. 6C, 3 and 5). Co-transfection with *Gbx2*Δ*HD* expression plasmid and the functional reporter did not result in an increase in transcriptional activity of the functional reporter gene, suggesting that the increase in activity requires the DNA-binding homeodomain of GBX2 (Fig. 6C, 3 and 6). Together, these data suggest that GBX2 acts synergistically through binding to the overlapping TATA/GBX2 binding sequence with other transcriptional regulators to provide maximal transcriptional activation of *EEF1A1*.

## Discussion

In this study we sought to gain further insight into the molecular mechanisms of *Gbx2*, by identifying cis-regulatory elements directly targeted by GBX2. Previous studies attempting to identify genes directly occupied by GBX2 have relied primarily on EMSA analysis providing a very limited number of target genes [36], [37]. This is the first report to utilize ChIP and high throughput sequencing technologies allowing an unbiased analysis of the complete genome to identify GBX2 target genes. This approach has revealed 286 candidate genes, which are expressed in a variety of tissues and have a diverse range of known functions (http://digbio.missouri.edu/gbx2/). We investigated several of the candidate genes identified through our bioinformatics analyses (*ROBO1*, *PCDH15*, *USH2A*, *NOTCH2,* and *EEF1A1*), and provide strong evidence through direct binding, co-expression, loss-of-expression and transcriptional activation studies supporting direct interaction with as well as regulation by GBX2.

### Regulation of target genes by GBX2

*Gbx2* is expressed in all three germ layers during gastrulation and dynamically throughout embryogenesis. Previously, gene inactivation studies of *Gbx2* have shown abnormal patterning and reduced levels of expression for many genes that could be downstream targets of GBX2 [7], [9], [10], [16]. However, as indicated by visualization through *in situ* analysis, gene expression of downstream targets is rarely completely down-regulated unless a region or tissue of the organism is absent. Thus, it is not clear if GBX2 functions primarily as an on/off switch or to modulate gene expression in the developing embryo. The vast majority of the target genes identified in this study are expressed in the nervous system, and could contribute to defects caused by loss-of *Gbx2* function. However, we unexpectedly found that most of the GBX2-bound target sequences are localized to introns: 4 out of 34 targets are within the first intron and 27 of the targets are contained in subsequent introns; 2 out of 34 targets are within exons. Surprisingly, only the target of *EEF1A1*, is located within the proximal promoter region. A possible explanation for the preponderance of intronic sequences is the use of PC-3 cells for the ChIP analysis. As such, the analysis is not exhaustive and almost certainly misses targets that may only be identified through an *in vivo* analysis.

It is well known that enhancers and repressors located in introns are bound by developmentally important transcription factors to mediate spatiotemporal control of gene expression without acting as direct on/off switches [62]–[64]. Our EMSA analysis showed that the target sequence of *ROBO1*, which is localized to the second intron, is directly occupied by GBX2. Furthermore, we showed through *in situ* hybridization that the expression of *Robo1* transcripts are markedly down-regulated in a subpopulation of NC cells within the r4 stream of *Gbx2^−/−^* mutants, resulting in a dramatic reduction of NC cells invading branchial arch 2. On the other hand, *Robo1* expression appears intact in other regions where Gbx2 is not expressed. While our observation is based on a single developmental time point (E9.5), it is in agreement with previous data showing that the initial NC cell migration and invasion of the branchial arches are distinct processes [65]. Moreover, it strongly suggests that *Gbx2* plays a critical role in promoting NC cell invasion through regulation of the Slit/Robo signaling pathway in a tissue-specific manner.

In addition to the numerous intronic sequences, our data show that GBX2 binds to the core promoter sequence of translation elongation factor *EEF1A1*. Notably, the target binding sequence (ATATAA) identified by MACS has significant overlap with the TATA box of *EEF1A1*. Several homeodomain-containing transcription factors, such as Engrailed (*En*) [66], [67] and even-skipped (*Eve*) [68], have been reported to bind to the TATA box and affect transcriptional activity. Similarly, CDX2 has been shown to repress the transcriptional activity of *Cabp9k* and *Shh* genes expressed in the small intestine and stomach, respectively, via binding to the TATA box [69], [70]. Together, these results indicate that transcription factors may interfere with transcription of the target genes by competing with the TATA box-binding protein transcription factor IID.

Based on loss-of-function and missexpression studies, *Gbx2* has been implicated as a repressor of *Otx2* expression at the MHB [7], [9]. Further studies using cell culture colocalization and coimmunoprecipitation experiments have demonstrated that GBX2 interacts with the WD40 domain of Groucho/Tle corepressor proteins through an Engrailed homology region 1 (eh-1) motif to repress Otx expression at the MHB [71]. In contrast, our results here show that GBX2 functions as a transcriptional activator of the *EEF1A1* gene. Our data indicate, that GBX2 functions as a transcriptional activator of EEF1A1, and particularly, through interaction with the core promoter sequence containing the TATA box. Several possibilities could account for these paradoxical results. Numerous factors contribute to DNA-protein binding and subsequent gene expression such as nucleotide sequences chromatin architecture and the presence of cofactors. First, it is well established that homeodomain proteins can participate in specific protein-protein interactions [72]. In addition to the eh-1 motif, GBX2 also has a proline rich region, which may contribute to protein-protein interactions. Therefore, it is possible that in addition to GBX2 DNA-binding through the homeodomain, maximal transcriptional regulation through the *EEF1A1* promoter requires its interaction with other proteins associated with the basal transcriptional machinery. This type of context dependent mechanism has been demonstrated in previous studies concerning other homeodomain proteins. The results from these studies demonstrate that transcriptional activity can be defined through the *in vitro* transcription system, actual function extends beyond DNA-binding [68], [69]. Secondly, it is possible that our parameters set for MACS (6 bp) did not allow for complete identification of the GBX2 binding site, and thus some key nucleotides involved with binding at either the 5′ or 3′ end of the identified sequence remain in the mutated non-functional expression construct allowing for GBX2 binding and activation. Lastly, chromatin modifications such as methylation and acetylation could in addition to DNA-binding by GBX2, play a role in whether transcriptional activation or repression occurs [62]. It is likely that no one particular, but a complex combination of these factors, contribute to regulation of *EEF1A1* by GBX2 in vivo. Nevertheless, our data presented in this study, along with previous reports support a role of GBX2 in modulating the spatiotemporal expression of its target genes rather than acting as a direct on/off switch.

Noteworthy, regulation of *EEF1A1* and potentially protein translation presents novel mechanisms through which GBX2 could function in development. This notion is supported by previous studies investigating the role of two members of the translation initiation factor 4A (eIF4A) family. The results from studies in *Xenopus* suggest that XeIF-4AIII, whose expression is upregulated in the ventral ectoderm of the gastrula embryo, interacts in a positive feedback loop with Bone Morphogenetic Protein-4 (BMP-4) in a positive feedback loop to induce epidermis and inhibit neural fate [73]. In contrast, eIF4AII is expressed specifically in the prospective neuroectoderm of *Xenopus* and upregulates several genes expressed early in the neural plate boarder and acts as a competence factor for neural induction [74]. Together, these studies provide examples of how eukaryotic translation elongation factors can contribute to cell fate decisions that determine neural fate. Therefore, additional studies aimed at investigating the regulation of *EEF1A1* by GBX2, may provide additional mechanisms by which GBX2 regulates neural development.

### GBX2 interacts with multiple genes associated with developmental disorders

Alterations in a number of proteins, including the cell-cell adhesion protein PCDH15 and transmembrane protein USH2A, are thought to directly contribute to the defects associated with Usher syndrome [75]. PCDH15 is associated with USH1F and is expressed along the length of the stereocilia and at the stereocilliary tip-links, the mechanosensitive gates to the ion channels [76]. Usherin (USH2A) is a large basement membrane protein and its expression at the base of developing hair bundles has been shown to be critical for their correct alignment and hair bundle formation [77]. Importantly, both proteins are required for correct formation of the stereocilia hair bundles in the inner ear and maintenance of normal cochlear and vestibular function. Studies of human cohorts have identified several single-base mutations within each gene as contributing to the manifestation of Usher 1 and 2 [78]. Similar to many mouse models for Usher syndrome, *Gbx2^−/−^* embryos display cochlear and vestibular sensory defects in the inner ear [30]. Interestingly, our data showed by RT-PCR that *Gbx2* is co-expressed with *Pcdh15* and *Ush2a* in both the vestibular and auditory components of the developing inner ear. More recent RNA-seq data have localized *Gbx2* expression to the vestibular hair cells and cochlear prosensory cells in mice (https://shield.harvard.hms.edu), which further support the notion of *Pcdh15* and *Ush2a* being direct targets of GBX2. Since GBX2 binds to intronic sequences for each of these genes it is likely to function in modulation of their expression rather than function as an on/off switch.

NC cells make an integral contribution to cardiovascular and craniofacial development. Indeed, several birth defects and congenital diseases are caused by abnormal NC cell development such as DiGeorge syndrome [34], [35], and craniofacial malformations. Interestingly, the first and second branchial arches represent critical niches in embryologic origin of many structures within the face [79]. We have identified several genes (*ROBO1*, *SLIT3*, and *NRP1*) that are involved in NC cell development as candidate target genes of GBX2. Recent studies have shown that *Nrp1* is expressed in migrating NC cells and along with *Nrp2* guide NC cells targeting branchial arch 2 [80]. We show here that *Robo1* expression is markedly down-regulated in the r4 stream of NC cells migrating into branchial arch 2 of *Gbx2^−/−^* embryos. Consistent with these findings, *Gbx2^−/−^* embryos display craniofacial and heart phenotypes resembling the clinical manifestation of these human defects. While our results suggest that GBX2 mediates NC cell migration, a recent study in *Xenopus* has shown that *Gbx2* is directly activated by Wnt/ß-catenin signaling during NC induction placing it as the earliest factor in the NC genetic cascade [81]. Intriguingly, results from this study show that *Gbx2* interacts with the neural fold gene *Zic1* for NC induction. Importantly, the expression of several NC genes including *Pax3*, *Snail2*, and *FoxD3*, were induced as a result of this interaction. Taken together, these data suggest that the loss of *Gbx2* may contribute to developmental disorders by impacting multiple facets of NC development.

In conclusion, our study has identified multiple candidate target genes of GBX2, which could directly contribute to many of the developmental defects observed in *Gbx2* mutant organisms. A knowledge of the mechanisms and signaling pathways regulated by *Gbx2* is important in understanding the abnormalities of *Gbx2* deficient organisms, which will in turn shed light onto multiple developmental disorders. While we have examined a few candidates here, further gene expression, DNA-binding and functional studies should provide a more comprehensive understanding of the molecular processes being affected due to deficiency in GBX2 expression. Insight into these processes could have broader implications towards understanding the etiology of multiple human genetic diseases and disorders.

## Materials and Methods

### Ethics Statement

The work performed in this manuscript is in compliance with the University of Missouri Office of Animal Care Quality Assurance (ACQA) under the protocol number 6479. The PC-3 cells were purchased from the ATCC (CRL-1435). No IRB approval is needed.

### Plasmid Constructs and ChIP Transfection

The full-length coding sequence for mouse *Gbx2* and a truncated *Gbx2* fragment omitting the homeobox, *Gbx2-*Δ*HD*, were subcloned into the pCMV-HA mammalian expression vector (Clontech). Plasmid constructs were verified by DNA sequencing using Gbx2 and pCMV-HA vector specific primers and analyzed using DNASTAR software. Human PC-3 cells from the ATCC (CRL-1435) were cultured in RPMI 1640 and supplemented with 2.5 µg/ml Amphotericin, Gentamicin, 2 mM L-Glutamine, 10 mM HEPES, 1 mM Sodium Pyruvate, 10% heat inactivated bovine serum and non-essential amino acids. One day prior to transfection, 8×10 cm dishes of PC-3 cells were seeded at a density of 3.5×10^6^ cells/10 cm dish. The next day, 4×10 cm dishes of PC-3 cells were transfected with either 7 µg of full-length *HA-Gbx2* or *HA-Gbx2*Δ*HD* plasmid with 21 µl of LipoD293 (Signagen Laboratories, Gaithersburg, MD) according to the manufacturer's directions.

### Immunoflouresence

Approximately, 3.5×10^5^ cells/35 mm PC-3 cells were seeded out. PC-3 cells were transfected with 2 µg of either full-length *HA-Gbx2* or *HA-Gbx2*Δ*HD* using 6 µl of LipoD293 according to the manufacturers directions. Cells were fixed and permeabilized 48 hours post transfection with 100% methanol and were covered with a 0.1% gelatin coated coverslip for 15 minutes at −20°C. The cells were washed with 1X PBS, pH 7.4. Cells were blocked with 10% fetal bovine serum and incubated with 1 µg of anti-HA antibody (Covance) for 30 minutes at room temperature. After washing with 1X PBS, pH 7.4, the cells were incubated with 4 µg Alexa Fluor488 (Invitrogen) and 100 ng nuclear staining Hoechst 33258 (Invitrogen) was applied. Cells were mounted on glass slides with Prolong Gold (Molecular Probes).

### ChIP-Seq and Bioinformatics Analysis

ChIP analysis on human PC-3 cells was conducted as described previously with some modifications [82]. Approximately, 3.5×10^6^ PC-3 cells were seeded out onto each of four ×10 cm dishes 24 hours prior to transfection. PC-3 cells were transfected with CMV transfection vectors encoding for either full-length HA-GBX2 or HA-GBX2ΔHD with LipoD293 (Signagen) according manufacturer's directions. At 40 hours post-transfection cells were crosslinked with 3.7% formaldehyde for 10 minutes at room temperature with gentle agitation. Crosslinking was stopped by the addition of 125 mm glycine/PBS. Cells were extensively washed with cold PBS, collected in cold PBS supplemented with protease inhibitors. Cell pellets were resuspended in cell lysis buffer (5 mM PIPES (KOH), pH 8.0, 85 mM KCl, 0.5% NP-40) supplemented with protease inhibitors lysed by dounce homogenization and the nuclei pelleted by centrifugation. Nuclei were resuspended in nuclear lysis buffer (50 mM Tris-Cl, pH 8.1, 10 mM EDTA, 1% SDS) supplemented with protease inhibitors. The chromatin was sonicated to approximately 300–600 bp. The debri was clarified by centrifugation and the supernatant containing the chromatin was diluted 5-fold in ChIP dilution buffer (0.01% SDS, 1.1% Triton X-100, 1.2 mM EDTA, 16.7 mM Tris-Cl, pH 8.1, 167 mM NaCl) supplemented with protease inhibitors. The chromatin was pre-cleared with a 50% *E. coli*. tRNA/1x PBS – protein A-agarose slurry for 1 hour at 4°C with gentle agitation. Twenty percent of the pre-cleared lysate was saved for a total input control. The remaining chromatin was left either untreated, treated with 5 ug anti-FLAG M2 (Sigma-Aldrich), treated with 10 ug anti-Histone H4 (Millipore), or treated with 5 µg of anti-HA.11 antibody (Covance). Immune Complexes were collected by the addition of 100 µl of a 50% *E. coli*. tRNA/1x PBS – protein A-agarose slurry at 4°C overnight and washed sequentially with a low salt buffer (0.1% SDS, 1% Triton X-100, 2 mM EDTA, 20 mM Tris, pH 8.1, 150 mM NaCl), high salt buffer (0.1% SDS, 1% Triton X-100, 2 mM EDTA, 20 mM Tris, pH 8.1, 500 mM NaCl), LiCl wash buffer (0.25 M LiCl, 1% NP40, 1% deoxycholate, 1 mM EDTA/10 mM Tris, pH 8.0) and twice in 1X TE buffer. Immune complexes were eluted by adding (2X) 250 µl of fresh elution buffer (1% SDS, 0.1 M NaHCO_3_) to the pelleted beads. The two elutions were combined and the formaldehyde crosslink's were reversed by adding 1 µl 10 mg/ml RNase and 5 M NaCl to a final concentration of 300 mM and incubating in a 65°C water bath for 5 hours. DNA was precipitated with 2.5 volumes of 100% ethanol at −20°C overnight. DNA was pelleted by centrifugation, resuspended in 100 µl water, 2 µl 0.5 M EDTA, 4 µl 1 M Tris, pH 6.5 and 1 µl of 20 mg/ml Proteinase K and incubated overnight at 45°C. DNA was purified using Qiaquick spin columns (Qiagen, Germantown, MD) and eluted in 20 µl sterile water.

Purified ChIP DNA and total input DNA were sequenced on the Illumina platform. Model-based Analysis of ChIP-Seq data (MACS) software was used to analyze the ChIP fragments from each of the sequenced samples [41]. Sequences were aligned to the hg18 build of the human genome on the UCSC Human Genome Browser [40]. GBX2 target genes were grouped together based on the tissues in which they are expressed using (DAVID) [42]. Enriched GBX2-bound sequence fragments were analyzed by Motif Sampler to identify GBX2 binding motifs [45].

### Electrophoretic Mobility Shift Assay

Synthesized oligonucleotides were annealed and end-labeled with [ÿ -^32^P] ATP. The sequences of the gel shift oligonucleotides can be found in Table S2. For the gel shift assay, 940 pM of labeled probe and 720 nM of GBX2 fusion protein were mixed and incubated with 2X binding buffer [20 mM Tris-HCL, pH 7.4, 50 mM KCL, 1 mM fresh dithiothreitol, 10% glycerol, 200 µg/mL BSA and 2.2 µg/mL Poly dI/dC] for 30 minutes at 25°C. The reactions were resolved on a 6% nondenaturing polyacrylamide gel at 300 v at room temperature using a 1X Tris-Glycine running buffer.

### RT-PCR

The membranous labyrinth of E13.5 inner ears were dissected, treated with thermolysin to remove surrounding mesenchyme, and separated into vestibular and auditory components without the endolymphatic duct [83]. Total RNA was extracted from these tissues using the RNeasy mini kit (Qiagen). First-strand cDNA was synthesized using SuperScript III Platinum Two-Step qRT-PCR Kit (Invitrogen) in accordance to the manufacturers instructions. After generation of cDNA, all samples were treated with RNase H at 37°C for 20 minutes. RT-PCR of *Gbx2* and identified targets were preformed by 40 cycles of PCR amplification using 3.0 µg cDNA generated from mouse inner ear tissues. Primer sequences can be found in Table S2. PCR conditions were determined empirically and products were resolved on 3% agarose gel containing ethidium bromide.

### Protein Expression in *E. coli* and Purification

The coding regions for *Gbx2* and *Gbx2ΔHD* were amplified by PCR and cloned into pET101/D-TOPO (Invitrogen). Primer sequences can be found in Table S2. *Gbx2*-coding sequences were verified by sequencing. Recombinant plasmids were transformed into *E.coli* BL21(DE3) (Invitrogen) and expressed fusion proteins purified using ProBond nickel-chelating resin (Invitrogen) in accordance to the manufacturer's instructions. The identity of the fusion proteins was confirmed by mass spectrometry.

### Mass Spectrometry

The major protein bands at approximately 13.5 kDa, 41 kDa and 50 kDa were excised from a coomassie blue-stained gel. The excised bands were destained, reduced with DTT, alkylated with iodoacetamide, and were trypsin digested overnight. The digested peptides were lyophilized to dryness and then resuspended in 60% CAN, 0.3% TFA. Peptides were mixed 1∶1 with CHCA matrix (5 mg/mL in 0.3% TFA, 60% ACN, and 10mM ammonium phosphate). Peptides were spotted onto the MALDI target and allowed to co-crystallize at room temperature. MS data were acquired with an Applied Biosystems 4700 Proteomics Analyzer instrument in positive ion mode with a mass range of 700 to 4000 Da. Then the top 8 precursor ions, excluding trypsin autolysis peptides, were selected for MS/MS analysis. Combined MS plus MS/MS search of NCBInr mammalian protein database was conducted with Applied Biosystems GPS Explorer V3.6 software as follows: ions with a signal/noise ratio greater than 20 (removing trypsin autolysis peptides) and a maximum of 125/spectrum were submitted against the NCBInr mammalian protein database (February 25^th^, 2008) using the MASCOT V2.1 search engine (www.matrixscience.com). The following search criteria were applied while using the MASCOT search engine: enzyme trypsin; 2 missed cleavages; carbamidomethyl cysteine; methionine oxidation; 100 ppm mass tolerance. The protein band at approximately 13.5 kDa was digested with trypsin followed by cyanogen bromide. This digest was then analyzed by LC-MS using an Agilent 1200 HPLC coupled to an Agilent 6520 Accurate Mass QTOF. An ion corresponding to the predicted SAAFPPSLM N-terminal CNBr-derived peptide was identified by mass in the data. Fragmentation spectra were then acquired on that peptide using a targeted MS/MS method, processed using qualitative analysis software (Agilent), and an MS/MS ion search of NCBInr limited to mammals conducted using MASCOT. Criteria for search were as follows: enzyme CNBr+trypsin; 1 missed cleavage; monoisotopic peptide mass; precursor ion mass error of 5 ppm; fragment ion mass error of 0.05 Da.

### Western Blot Analysis

For Western blot detection of HA-GBX2 and HA-GBX2ΔHD, 32.4 µg of total RIPA lystates, 15 µl of immunoprecipitated protein lysates, and 765 ng of full-length GBX2 and 1.3 µg or GBX2ΔHD were resolved on 10% SDS-polyacrylamide gels. Proteins were electroblotted onto Whatman Protran BA 83 nitrocellulose (Dassel, Germany). The membranes were rinsed for 5 minutes with 1x TTBS (140 mM NaCl, 2.7 mM KCL, 0.1% Tween 20, 25 mM Tris, pH 7.5) and blocked for 30 minutes in 3% milk in 1X TTBS. After removing the blocking solution, membranes were incubated with primary antibody, mouse monoclonal anti-HA.11 (Covance, Emeryville, California) for HA-GBX2, horseradish peroxidase-conjugated anti-V5 (Invitrogen, Carlsbad, CA) or anti-Gbx2, (Novus Biologicals, Littleton, CO) for full-length recombinant mouse GBX2 and GBX2ΔHD in 1% milk in 1X TTBS for 3 hours at room temperature. After washing, the membranes were incubated with horseradish peroxidase-conjugated horse anti-mouse IgG linked antibody (Cell Signaling), or horseradish peroxidase-conjugated rabbit anti-goat IgG F(ab')_2_ (Jackson ImmunoResearch Laboratories, West Grove, PA). After washing, membranes were incubated with ECL plus (Amersham Biosciences, Pittsburg, PA) and protein complexes were visualized by chemiluminescence.

### Luciferase Reporter Assays

To generate our reporter constructs, *EEF1A1* oligos containing the functional and mutated promoter were annealed together. Functional *EEF1A1* annealed oligos were digested with *KpnI* and *XhoI*. The digested oligos were column purified using Qiaquick spin columns (Qiagen, Germantown, MD) and eluted in 20 µl water. Functional and mutated *EEF1A1* promoter oligos were cloned into the KpnI and XhoI sites of the pGL4.10[*luc2*] vector (Promega) and confirmed by sequencing using DNASTAR software. On the day prior to transfection, 1.5×10^5^ human embryonic kidney (HEK) 293FT cells were seeded out on 24-well plates and cultured in DMEM-HG (Invitrogen) supplemented with 2 mM L-glutamine, 10% (vol/vol) fetal bovine serum, 0.05 µg/ml gentamicin, 2.5 µg/ml Amphotericin, 100 U/ml penicillin, and 100 mg/ml streptomycin at 37°C in 4.0% CO_2_. Transient transfections were preformed using LipoD293 transfection reagents according to the manufacture directions (Signagen Laboratories, Gaithersburg, MD). Cells were transiently transfected with 50 ng of the *Renilla* luciferase reporter plasmid, and 500 ng of either empty pGL4.10[*luc2*], functional, or mutated *EEF1A1* promoter reporter plasmids. In addition, cells were also transiently transfected with empty *pCMV-HA* vector, *HA-Gbx2*, or *HA-Gbx2*Δ*HD*. After the transfections, cells were maintained for 48 hours before they were lysed in 1X lysis buffer (Promega). The *firefly* luciferase activity was normalized to the *Renilla* luciferase activity and both luciferase activities were analyzed using the Dual-Luciferase Reporter Assay (Promega). Experiments were preformed three times in triplicate.

### In situ hybridizations

In situ hybridizations were performed as previously described [3].

## Supporting Information

Figure S1 Co-expression of *Gbx2* and *Robo1* at E9.5 in wild-type embryos. (A–E) Whole mount in situ hybridization for *Gbx2* (A,B) and *Gbx2/Robo1* (C–E). (A lateral view, B dorsal view) *Gbx2* is highly expressed in regions in which neural crest contributes, or migrates from, such as the otic vesicle, branchial arch 1 and the longitudinal columns (arrows) flanking r4. (C–E) shows co-expression of *Gbx2* and *Robo1* in regions populated by neural crest. (C) lateral view showing co-expression of Robo1 (purple) and Gbx2 (orange). (D) transverse image of section through (C) indicated by long line indicates co-expression of Gbx2 and Robo1 in branchial arch 1. (E) transverse image of section through (C) indicated by short line indicates co-expression of *Gbx2* and *Robo1* in the otic vesicle. ba1  =  branchial arch 1; ov  =  otic vesicle.
(TIF)

Table S1 The top 286 ChIP-Seq identified GBX2 targets. The top 286 genes targeted by GBX2 in human PC-3 cells based on ChIP-Seq fragments aligned to the hg18 build of the human genome on the UCSC Human Genome Browser. The top 286 GBX2 target genes are organized by the P values.
(DOC)

Table S2 Primer and oligonucleotide sequences used for identifying and analyzing GBX2 target genes. Data table containing the primer and oligonucleotide sequences used for identifying and analyzing GBX2 target genes. Bold type indicates GBX2 binding sites.
(DOC)
