# Supplementary figures and images for: Elongation Factor 1 alpha1 and Genes Associated with Usher Syndromes Are Downstream Targets of GBX2
Source: PLoS One. 2012 Nov 8;7(11):e47366. doi: 10.1371/journal.pone.0047366 (PMC3493575; doi:10.1371/journal.pone.0047366)

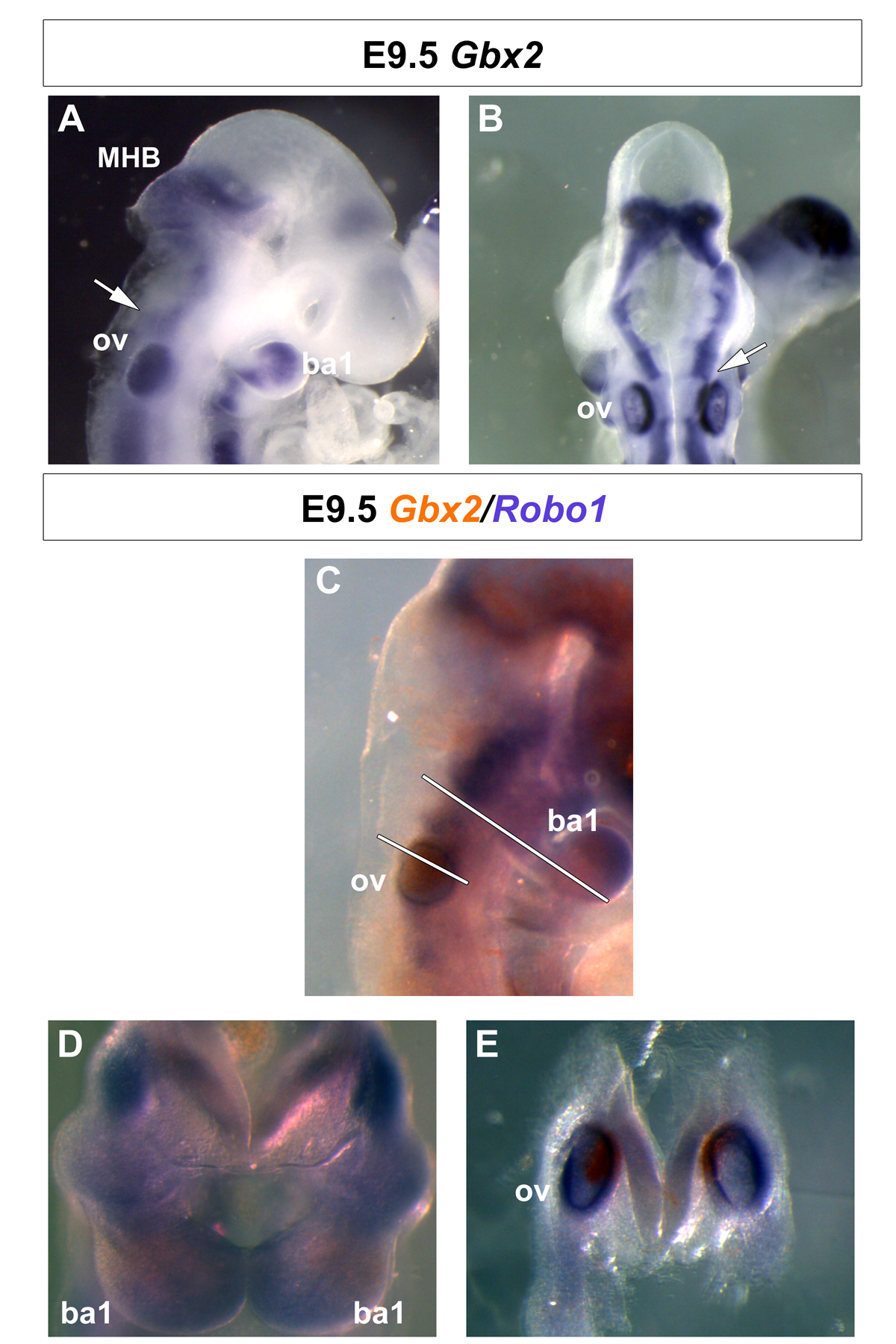

Supplement: Figure S1 — Co-expression of Gbx2 and Robo1 at E9.5 in wild-type embryos. (A–E) Whole mount in situ hybridization for Gbx2 (A,B) and Gbx2/Robo1 (C–E). (A lateral view, B dorsal view) Gbx2 is highly expressed in regions in which neural crest contributes, or migrates from, such as the otic vesicle, branchial arch 1 and the longitudinal columns (arrows) flanking r4. (C–E) shows co-expression of Gbx2 and Robo1 in regions populated by neural crest. (C) lateral view showing co-expression of Robo1 (purple) and Gbx2 (orange). (D) transverse image of section through (C) indicated by long line indicates co-expression of Gbx2 and Robo1 in branchial arch 1. (E) transverse image of section through (C) indicated by short line indicates co-expression of Gbx2 and Robo1 in the otic vesicle. ba1 = branchial arch 1; ov = otic vesicle. (TIF) [file pone.0047366.s001.tif]
