# Supplementary material for: Elongation Factor 1 alpha1 and Genes Associated with Usher Syndromes Are Downstream Targets of GBX2
Source: PLoS One. 2012 Nov 8;7(11):e47366. doi: 10.1371/journal.pone.0047366 (PMC3493575; doi:10.1371/journal.pone.0047366)
Supplement: Table S1 — The top 286 ChIP-Seq identified GBX2 targets. The top 286 genes targeted by GBX2 in human PC-3 cells based on ChIP-Seq fragments aligned to the hg18 build of the human genome on the UCSC Human Genome Browser. The top 286 GBX2 target genes are organized by the P values. (DOC) [file pone.0047366.s002.doc]

Supplementary Table S1. The top 286 ChIP-Seq identified GBX2 targets.

| **Candidate Gene ID** | **P value** | **Fold enrichment** | **Numberof tags** | **Gene Name** |
| --- | --- | --- | --- | --- |
| NFE2L2 | 2.6 X 10^-270^ | 26.18 | 376 | NUCLEAR FACTOR (ERYTHROID-DERIVED 2)-LIKE 2 |
| LOC100130885 | 2.6 X 10^-270^ | 26.18 | 376 |  |
| LOC100129409 | 1.2 X 10^-36^ | 6.26 | 154 |  |
| EEF1A1 | 1.2 X 10^-36^ | 6.26 | 154 | EUKARYOTIC TRANSLATION ELONGATION FACTOR 1 ALPHA 1 |
| FLJ20433 | 5.5 X 10^-15^ | 38.58 | 13 | HYPOTHETICAL PROTEIN FLJ20433 |
| OTUD7B | 4.1 X 10^-13^ | 90.17 | 8 | ZINC FINGER, A20 DOMAIN CONTAINING 1 |
| ULK4 | 1.2 X 10^-12^ | 80.17 | 8 | UNC-51-LIKE KINASE 4 (C. ELEGANS) |
| OR5H1 | 3.0 X 10^-10^ | 12.72 | 19 | OLFACTORY RECEPTOR, FAMILY 5, SUBFAMILY H, MEMBER 1 |
| HAO2 | 8.1 X 10^-9^ | 41.34 | 6 | HYDROXYACID OXIDASE 2 (LONG CHAIN) |
| DPY19L2P2 | 5.4 X 10^-9^ | 33.81 | 6 | HYPOTHETICAL PROTEIN FLJ36166 |
| ACSL1 | 2.9 X 10^-9^ | 45.09 | 6 | FATTY-ACID-COENZYME A LIGASE, LONG-CHAIN 1 |
| LOC100132858 | 2.8 X 10^-9^ | 11.31 | 14 |  |
| ELMO1 | 2.4 X 10^-9^ | 42.87 | 7 | ENGULFMENT AND CELL MOTILITY 1 |
| MAL2 | 1.7 X 10^-9^ | 62 | 6 | MAL, T-CELL DIFFERENTIATION PROTEIN 2 |
| SNAP25 | 9.8 X 10^-8^ | 34.81 | 6 | SYNAPTOSOMAL-ASSOCIATED PROTEIN, 25KDA |
| HLF | 9.7 X 10^-8^ | 41.34 | 5 | HEPATIC LEUKEMIA FACTOR |
| SOCS4 | 9.7 X 10^-8^ | 45.09 | 5 | SUPPRESSOR OF CYTOKINE SIGNALING 4 |
| WDSUB1 | 8.9 X 10^-8^ | 33.81 | 5 | WD REPEAT, STERILE ALPHA MOTIF AND U-BOX DOMAIN CONTAINING 1 |
| PPP3CB | 8.9 X 10^-8^ | 18.52 | 9 | PROTEIN PHOSPHATASE 3 (FORMERLY 2B), CATALYTIC SUBUNIT, BETA ISOFORM (CALCINEURIN A BETA) |
| FREM2 | 7.6 X 10^-8^ | 33.81 | 5 | FRAS1 RELATED EXTRACELLULAR MATRIX PROTEIN 2 |
| LAMA2 | 6.2 X 10^-8^ | 51.67 | 5 | LAMININ, ALPHA 2 (MEROSIN, CONGENITAL MUSCULAR DYSTROPHY) |
| PLXNA4 | 4.4 X 10^-8^ | 33.07 | 6 | PLEXIN A4, B |
| LOC644496 | 4.3 X 10^-8^ | 19.84 | 8 | SIMILAR TO CATHEPSIN L PRECURSOR (MAJOR EXCRETED PROTEIN) (MEP) |
| CNBD1 | 4.0 X 10^-8^ | 40.33 | 6 | CYCLIC NUCLEOTIDE BINDING DOMAIN CONTAINING 1 |
| ABCA9 | 3.7 X 10^-8^ | 56.36 | 5 | ATP-BINDING CASSETTE, SUB-FAMILY A (ABC1), MEMBER 9 |
| ABCC3 | 3.7 X 10^-8^ | 56.36 | 5 | ATP-BINDING CASSETTE, SUB-FAMILY C (CFTR/MRP), MEMBER 3 |
| B3GAT2 | 3.7 X 10^-8^ | 45.09 | 5 | BETA-1,3-GLUCURONYLTRANSFERASE 2 (GLUCURONOSYLTRANSFERASE S) |
| DDHD1 | 3.7 X 10^-8^ | 56.36 | 5 | DDHD DOMAIN CONTAINING 1 |
| ERC2 | 3.7 X 10^-8^ | 56.36 | 5 | CAZ-ASSOCIATED STRUCTURAL PROTEIN |
| FAM19A1 | 3.7 X 10^-8^ | 56.36 | 5 | FAMILY WITH SEQUENCE SIMILARITY 19 (CHEMOKINE (C-C MOTIF)-LIKE), MEMBER A1 |
| LOC653269 | 3.7 X 10^-8^ | 56.36 | 5 | SIMILAR TO PROTEIN EXPRESSED IN PROSTATE, OVARY, TESTIS, AND PLACENTA 15 |
| PTPRD | 3.7 X 10^-8^ | 56.36 | 5 |  |
| ROBO1 | 3.7 X 10^-8^ | 56.36 | 5 | ROUNDABOUT, AXON GUIDANCE RECEPTOR, HOMOLOG 1 (DROSOPHILA) |
| SCFD2 | 3.7 X 10^-8^ | 56.36 | 5 | SEC1 FAMILY DOMAIN CONTAINING 2 |
| SLC16A7 | 3.7 X 10^-8^ | 56.36 | 5 | SOLUTE CARRIER FAMILY 16 (MONOCARBOXYLIC ACID TRANSPORTERS), MEMBER 7 |
| TGFA | 3.7 X 10^-8^ | 45.09 | 5 | TRANSFORMING GROWTH FACTOR, ALPHA |
| TMEM16K | 3.7 X 10^-8^ | 56.36 | 5 | TRANSMEMBRANE PROTEIN 16K |
| TNFRSF21 | 3.7 X 10^-8^ | 56.36 | 5 | TUMOR NECROSIS FACTOR RECEPTOR SUPERFAMILY, MEMBER 21 |
| GUSBL1 | 3.5 X 10^-8^ | 39.68 | 6 | SMA4 |
| LOC729400 | 3.5 X 10^-8^ | 39.68 | 6 |  |
| VCL | 2.1 X 10^-8^ | 24.8 | 7 | VINCULIN |
| SYT2 | 1.7 X 10^-8^ | 27.56 | 7 | SYNAPTOTAGMIN II |
| ADNP | 1.6 X 10^-8^ | 33.81 | 6 | ACTIVITY-DEPENDENT NEUROPROTECTOR |
| LOC100131754 | 1.2 X 10^-8^ | 8.97 | 21 |  |
| LOC348808 | 1.1 X 10^-8^ | 43.51 | 6 | BC048124 |
| LOC100132518 | 1.1 X 10^-8^ | 22.05 | 7 |  |
| GPHN | 9.8 X 10^-7^ | 24.8 | 5 | GEPHYRIN |
| LOC100133326 | 9.8 X 10^-7^ | 19.84 | 5 |  |
| LOC727978 | 9.8 X 10^-7^ | 19.84 | 5 |  |
| PTPRR | 9.8 X 10^-7^ | 24.8 | 5 | PROTEIN TYROSINE PHOSPHATASE, RECEPTOR TYPE, R |
| LOC402117 | 9.8 X 10^-7^ | 33.92 | 5 | HYPOTHETICAL LOC402117 |
| SLC10A7 | 9.7 X 10^-7^ | 26.11 | 5 | CHROMOSOME 4 OPEN READING FRAME 13 |
| CCNY | 9.2 X 10^-7^ | 12.4 | 8 | CHROMOSOME 10 OPEN READING FRAME 9 |
| RTN1 | 9.2 X 10^-7^ | 15.34 | 8 | RETICULON 1 |
| TIMD4 | 9.2 X 10^-7^ | 13.89 | 8 | T-CELL IMMUNOGLOBULIN AND MUCIN DOMAIN CONTAINING 4 |
| PARD3 | 9.0 X 10^-7^ | 12.02 | 10 | PAR-3 PARTITIONING DEFECTIVE 3 HOMOLOG (C. ELEGANS) |
| MLZE | 8.8 X 10^-7^ | 30.76 | 5 | MELANOMA-DERIVED LEUCINE ZIPPER, EXTRA-NUCLEAR FACTOR |
| HS6ST3 | 8.5 X 10^-7^ | 26.45 | 6 | HEPARAN SULFATE 6-O-SULFOTRANSFERASE 3 |
| LOC391764 | 8.5 X 10^-7^ | 33.07 | 5 | SIMILAR TO TAF11 RNA POLYMERASE II, TATA BOX BINDING PROTEIN (TBP)-ASSOCIATED FACTOR |
| NPAS3 | 8.3 X 10^-7^ | 27.56 | 6 | NEURONAL PAS DOMAIN PROTEIN 3 |
| FAT3 | 8.3 X 10^-7^ | 21 | 6 | FAT TUMOR SUPPRESSOR HOMOLOG 3 (DROSOPHILA) |
| AGT | 8.2 X 10^-7^ | 33.07 | 5 | ANGIOTENSINOGEN (SERPIN PEPTIDASE INHIBITOR, CLADE A, MEMBER 8) |
| COL14A1 | 8.2 X 10^-7^ | 26.45 | 5 | COLLAGEN, TYPE XIV, ALPHA 1 (UNDULIN) |
| DGKB | 8.2 X 10^-7^ | 33.07 | 5 | DIACYLGLYCEROL KINASE, BETA 90KDA |
| FGF12 | 8.2 X 10^-7^ | 13.23 | 9 | FIBROBLAST GROWTH FACTOR 12 |
| ZNF350 | 8.2 X 10^-7^ | 33.07 | 5 | ZINC FINGER PROTEIN 350 |
| LOC338805 | 7.6 X 10^-7^ | 27.56 | 5 | SIMILAR TO HEAT SHOCK 70KD PROTEIN BINDING PROTEIN |
| FLJ46266 | 6.7 X 10^-7^ | 29.18 | 5 | HYPOTHETICAL GENE SUPPORTED BY BC039505 |
| A2BP1 | 6.5 X 10^-7^ | 34.45 | 5 | ATAXIN 2-BINDING PROTEIN 1 |
| KIAA1009 | 6.3 X 10^-7^ | 24.8 | 5 | CHROMOSOME 6 OPEN READING FRAME 84 |
| LOC100130590 | 6.3 X 10^-7^ | 24.8 | 5 |  |
| LOC729920 | 6.3 X 10^-7^ | 24.8 | 5 |  |
| MLL3 | 6.3 X 10^-7^ | 24.8 | 5 | MYELOID/LYMPHOID OR MIXED-LINEAGE LEUKEMIA 3 |
| CSPP1 | 5.8 X 10^-7^ | 24.8 | 5 | CENTROSOME AND SPINDLE POLE ASSOCIATED PROTEIN 1 |
| SRGAP2L | 5.5 X 10^-7^ | 22.96 | 6 |  |
| FBLN5 | 5.4 X 10^-7^ | 27.56 | 5 | FIBULIN 5 |
| LOC100129904 | 5.4 X 10^-7^ | 24.8 | 5 |  |
| LOC730234 | 5.4 X 10^-7^ | 24.8 | 5 |  |
| LOC730235 | 5.4 X 10^-7^ | 24.8 | 5 |  |
| NRP1 | 5.3 X 10^-7^ | 15.03 | 7 | NEUROPILIN 1 |
| LOC100132615 | 5.1 X 10^-7^ | 18.9 | 8 |  |
| HRSP12 | 4.8 X 10^-7^ | 32 | 5 | HEAT-RESPONSIVE PROTEIN 12 |
| SSPN | 4.6 X 10^-7^ | 33.07 | 5 | SARCOSPAN (KRAS ONCOGENE-ASSOCIATED GENE) |
| DNM3 | 4.5 X 10^-7^ | 36.74 | 5 | DYNAMIN 3 |
| LOC100128178 | 4.5 X 10^-7^ | 36.74 | 5 |  |
| NPSR1 | 4.5 X 10^-7^ | 36.74 | 5 | G PROTEIN-COUPLED RECEPTOR 154 |
| NR5A2 | 4.4 X 10^-7^ | 31 | 5 | NUCLEAR RECEPTOR SUBFAMILY 5, GROUP A, MEMBER 2 |
| PTK2 | 4.3 X 10^-7^ | 34.81 | 5 | PTK2 PROTEIN TYROSINE KINASE 2 |
| SRGAP3 | 4.0 X 10^-7^ | 30.06 | 5 | SLIT-ROBO RHO GTPASE ACTIVATING PROTEIN 3 |
| JAKMIP2 | 3.9 X 10^-7^ | 41.34 | 5 | JANUS KINASE AND MICROTUBULE INTERACTING PROTEIN 2 |
| LOC153469 | 3.9 X 10^-7^ | 41.34 | 5 | HYPOTHETICAL PROTEIN LOC153469 |
| LCP2 | 3.5 X 10^-7^ | 21.57 | 6 | LYMPHOCYTE CYTOSOLIC PROTEIN 2 (SH2 DOMAIN CONTAINING LEUKOCYTE PROTEIN OF 76KDA) |
| DPP10 | 3.5 X 10^-7^ | 27.56 | 6 | DIPEPTIDYL-PEPTIDASE 10 |
| FAM19A4 | 3.5 X 10^-7^ | 27.56 | 6 | FAMILY WITH SEQUENCE SIMILARITY 19 (CHEMOKINE (C-C MOTIF)-LIKE), MEMBER A4 |
| NCOA2 | 3.5 X 10^-7^ | 33.81 | 5 | NUCLEAR RECEPTOR COACTIVATOR 2 |
| CCDC85A | 3.3 X 10^-7^ | 45.09 | 5 | KIAA1912 PROTEIN |
| CTGLF10P | 3.3 X 10^-7^ | 33.07 | 5 | SIMILAR TO CENTAURIN, GAMMA-LIKE FAMILY, MEMBER 1 |
| FGF7P2 | 3.2 X 10^-7^ | 27.56 | 6 | FIBROBLAST GROWTH FACTOR 7-LIKE |
| SRPK2 | 3.0 X 10^-7^ | 33.81 | 5 | SFRS PROTEIN KINASE 2 |
| LOC400986 | 3.0 X 10^-7^ | 26.67 | 6 | PROTEIN IMMUNO-REACTIVE WITH ANTI-PTH POLYCLONAL ANTIBODIES |
| MARCH4 | 3.0 X 10^-7^ | 44.09 | 5 |  |
| PCDH7 | 3.0 X 10^-7^ | 44.09 | 5 | BH-PROTOCADHERIN (BRAIN-HEART) |
| FLJ45872 | 3.0 X 10^-7^ | 12.97 | 9 | FLJ45872 PROTEIN |
| BAI3 | 2.9 X 10^-7^ | 33.81 | 5 | BRAIN-SPECIFIC ANGIOGENESIS INHIBITOR 3 |
| C11orf80 | 2.9 X 10^-7^ | 45.09 | 5 | HYPOTHETICAL PROTEIN FLJ22531 |
| DSE | 2.9 X 10^-7^ | 45.09 | 5 | SQUAMOUS CELL CARCINOMA ANTIGEN RECOGNIZED BY T CELLS 2 |
| FBXO11 | 2.9 X 10^-7^ | 33.81 | 5 | F-BOX ONLY PROTEIN 11 |
| FUT8 | 2.9 X 10^-7^ | 33.81 | 5 | FUCOSYLTRANSFERASE 8 (ALPHA (1,6) FUCOSYLTRANSFERASE) |
| LIN9 | 2.9 X 10^-7^ | 33.81 | 5 | LIN-9 HOMOLOG (C. ELEGANS) |
| MMP16 | 2.9 X 10^-7^ | 33.81 | 5 | MATRIX METALLOPEPTIDASE 16 (MEMBRANE-INSERTED) |
| LOC344595 | 2.7 X 10^-7^ | 36.74 | 5 | HYPOTHETICAL LOC344595 |
| CLASP2 | 2.7 X 10^-7^ | 33.81 | 5 | CYTOPLASMIC LINKER ASSOCIATED PROTEIN 2 |
| CNIH | 2.7 X 10^-7^ | 45.09 | 5 | CORNICHON HOMOLOG (DROSOPHILA) |
| MYLK | 2.7 X 10^-7^ | 33.81 | 5 | MYOSIN, LIGHT POLYPEPTIDE KINASE |
| OR13K1P | 2.7 X 10^-7^ | 33.81 | 5 | OLFACTORY RECEPTOR, FAMILY 13, SUBFAMILY K, MEMBER 1 PSEUDOGENE |
| RNF150 | 2.7 X 10^-7^ | 38.9 | 5 | RING FINGER PROTEIN 150 |
| VPS8 | 2.7 X 10^-7^ | 33.07 | 5 | KIAA0804 |
| GMDS | 2.5 X 10^-7^ | 33.81 | 5 | GDP-MANNOSE 4,6-DEHYDRATASE |
| SLC22A15 | 2.5 X 10^-7^ | 33.81 | 5 | SOLUTE CARRIER FAMILY 22 (ORGANIC CATION TRANSPORTER), MEMBER 15 |
| UBR4 | 2.5 X 10^-7^ | 33.81 | 5 | ZINC FINGER, UBR1 TYPE 1 |
| IMMT | 2.5 X 10^-7^ | 33.07 | 5 | INNER MEMBRANE PROTEIN, MITOCHONDRIAL (MITOFILIN) |
| SLC38A6 | 2.5 X 10^-7^ | 33.07 | 5 | SOLUTE CARRIER FAMILY 38, MEMBER 6 |
| C6orf142 | 2.3 X 10^-7^ | 33.81 | 5 | CHROMOSOME 6 OPEN READING FRAME 142 |
| LOC653188 | 2.3 X 10^-7^ | 41.34 | 5 | SIMILAR TO BETA-GLUCURONIDASE PRECURSOR |
| PDE11A | 2.3 X 10^-7^ | 18.37 | 8 | PHOSPHODIESTERASE 11A |
| CCBE1 | 2.2 X 10^-7^ | 30.06 | 5 | COLLAGEN AND CALCIUM BINDING EGF DOMAINS 1 |
| ANK3 | 2.2 X 10^-7^ | 41.34 | 5 | ANKYRIN 3, NODE OF RANVIER (ANKYRIN G) |
| LOC728806 | 2.2 X 10^-7^ | 45.09 | 5 |  |
| SAMD8 | 2.1 X 10^-7^ | 23.62 | 6 | STERILE ALPHA MOTIF DOMAIN CONTAINING 8 |
| DHRSX | 2.0 X 10^-7^ | 33.81 | 5 | DEHYDROGENASE/REDUCTASE (SDR FAMILY) X-LINKED |
| PCNX | 2.0 X 10^-7^ | 33.81 | 5 | PECANEX HOMOLOG (DROSOPHILA) |
| PVT1 | 2.0 X 10^-7^ | 33.81 | 5 | PVT1 ONCOGENE HOMOLOG, MYC ACTIVATOR (MOUSE) |
| LOC441416 | 2.0 X 10^-7^ | 42.4 | 5 | SIMILAR TO G-PROTEIN COUPLED RECEPTOR 116 |
| BRE | 1.9 X 10^-7^ | 45.09 | 5 | BRAIN AND REPRODUCTIVE ORGAN-EXPRESSED (TNFRSF1A MODULATOR) |
| ICAM3 | 1.9 X 10^-7^ | 33.81 | 5 | INTERCELLULAR ADHESION MOLECULE 3 |
| SNF1LK2 | 1.9 X 10^-7^ | 45.09 | 5 | SNF1-LIKE KINASE 2 |
| LOC729669 | 1.9 X 10^-7^ | 33.07 | 5 |  |
| LOC100128714 | 1.8 X 10^-7^ | 35.18 | 6 |  |
| SBF2 | 1.8 X 10^-7^ | 45.09 | 5 | SET BINDING FACTOR 2 |
| SFMBT2 | 1.8 X 10^-7^ | 45.09 | 5 | SCM-LIKE WITH FOUR MBT DOMAINS 2 |
| ADK | 1.7 X 10^-7^ | 21.33 | 6 | ADENOSINE KINASE |
| GRB14 | 1.5 X 10^-7^ | 33.81 | 5 | GROWTH FACTOR RECEPTOR-BOUND PROTEIN 14 |
| KIAA1333 | 1.5 X 10^-7^ | 45.09 | 5 | KIAA1333 |
| GPR128 | 1.5 X 10^-7^ | 44.09 | 5 | G PROTEIN-COUPLED RECEPTOR 128 |
| NEB | 1.4 X 10^-7^ | 33.81 | 5 | NEBULIN |
| PRKAR2B | 1.4 X 10^-7^ | 33.07 | 6 | PROTEIN KINASE, CAMP-DEPENDENT, REGULATORY, TYPE II, BETA |
| JMJD1C | 1.3 X 10^-7^ | 36.74 | 5 | THYROID HORMONE RECEPTOR INTERACTOR 8 |
| SLAMF1 | 1.3 X 10^-7^ | 45.09 | 5 | SIGNALING LYMPHOCYTIC ACTIVATION MOLECULE FAMILY MEMBER 1 |
| HIF1A | 1.3 X 10^-7^ | 26.45 | 6 | HYPOXIA-INDUCIBLE FACTOR 1, ALPHA SUBUNIT (BASIC HELIX-LOOP-HELIX TRANSCRIPTION FACTOR) |
| PGCP | 1.3 X 10^-7^ | 33.07 | 6 | PLASMA GLUTAMATE CARBOXYPEPTIDASE |
| GRIK3 | 1.2 X 10^-7^ | 31.49 | 6 | GLUTAMATE RECEPTOR, IONOTROPIC, KAINATE 3 |
| LOC100129460 | 1.2 X 10^-7^ | 45.93 | 5 |  |
| IL1RAP | 1.2 X 10^-7^ | 45.09 | 5 | INTERLEUKIN 1 RECEPTOR ACCESSORY PROTEIN |
| PTPN14 | 1.2 X 10^-7^ | 33.81 | 5 | PROTEIN TYROSINE PHOSPHATASE, NON-RECEPTOR TYPE 14 |
| GALNTL4 | 1.1 X 10^-7^ | 45.09 | 5 | UDP-N-ACETYL-ALPHA-D-GALACTOSAMINE:POLYPEPTIDE N-ACETYLGALACTOSAMINYLTRANSFERASE-LIKE 4 |
| NSMCE2 | 1.1 X 10^-7^ | 36.74 | 6 | CHROMOSOME 8 OPEN READING FRAME 36 |
| MYST4 | 1.1 X 10^-7^ | 22.05 | 7 | MYST HISTONE ACETYLTRANSFERASE (MONOCYTIC LEUKEMIA) 4 |
| PI15 | 9.9 X 10^-6^ | 18.37 | 5 | PEPTIDASE INHIBITOR 15 |
| BTBD11 | 9.8 X 10^-6^ | 16.53 | 5 | BTB (POZ) DOMAIN CONTAINING 11 |
| NUBPL | 9.8 X 10^-6^ | 16.53 | 5 | NUCLEOTIDE BINDING PROTEIN-LIKE |
| SYT14 | 9.8 X 10^-6^ | 16.53 | 5 | SYNAPTOTAGMIN XIV |
| MACROD2 | 9.1 X 10^-6^ | 16.53 | 5 | CHROMOSOME 20 OPEN READING FRAME 133 |
| PRDM14 | 9.1 X 10^-6^ | 22.05 | 5 | PR DOMAIN CONTAINING 14 |
| SGMS1 | 9.1 X 10^-6^ | 16.53 | 5 | TRANSMEMBRANE PROTEIN 23 |
| NOTCH2 | 9.1 X 10^-6^ | 14.7 | 6 | NOTCH HOMOLOG 2 (DROSOPHILA) |
| FAM21C | 9.0 X 10^-6^ | 18.04 | 5 | KIAA0592 PROTEIN |
| CBWD2 | 8.6 X 10^-6^ | 19.45 | 5 |  |
| LOC646870 | 8.6 X 10^-6^ | 19.45 | 5 | HYPOTHETICAL PROTEIN LOC646870 |
| DAB1 | 8.6 X 10^-6^ | 18.9 | 5 | DISABLED HOMOLOG 1 (DROSOPHILA) |
| PTN | 8.6 X 10^-6^ | 14.81 | 5 | PLEIOTROPHIN (HEPARIN BINDING GROWTH FACTOR 8, NEURITE GROWTH-PROMOTING FACTOR 1) |
| CDH13 | 8.6 X 10^-6^ | 13.23 | 7 | CADHERIN 13, H-CADHERIN (HEART) |
| CEP170 | 8.6 X 10^-6^ | 13.76 | 7 | CENTROSOMAL PROTEIN 170KDA |
| LOC349114 | 8.6 X 10^-6^ | 18.02 | 7 | HYPOTHETICAL PROTEIN LOC349114 |
| LOC727726 | 8.6 X 10^-6^ | 11.77 | 7 |  |
| LOC728297 | 8.6 X 10^-6^ | 11.57 | 7 |  |
| PCSK2 | 8.6 X 10^-6^ | 14.29 | 7 | PROPROTEIN CONVERTASE SUBTILISIN/KEXIN TYPE 2 |
| ADCY8 | 8.5 X 10^-6^ | 7.19 | 10 | ADENYLATE CYCLASE 8 (BRAIN) |
| KCNMA1 | 8.5 X 10^-6^ | 22.05 | 5 | POTASSIUM LARGE CONDUCTANCE CALCIUM-ACTIVATED CHANNEL, SUBFAMILY M, ALPHA MEMBER 1 |
| LOC100132617 | 8.5 X 10^-6^ | 22.05 | 5 |  |
| TPK1 | 8.5 X 10^-6^ | 18.04 | 5 | THIAMIN PYROPHOSPHOKINASE 1 |
| WDR49 | 8.4 X 10^-6^ | 15.5 | 5 | WD REPEAT DOMAIN 49 |
| LOC100129561 | 8.4 X 10^-6^ | 14.7 | 6 |  |
| LOC642446 | 8.3 X 10^-6^ | 15.75 | 5 | SIMILAR TO TRIPARTITE MOTIF PROTEIN 17 |
| PRKG1 | 8.2 X 10^-6^ | 17.64 | 5 | PROTEIN KINASE, CGMP-DEPENDENT, TYPE I |
| LOC285900 | 8.2 X 10^-6^ | 23.62 | 5 | SIMILAR TO 60S RIBOSOMAL PROTEIN L6 (TAX-RESPONSIVE ENHANCER ELEMENT BINDING PROTEIN 107) (TAXREB107) (NEOPLASM-RELATED PROTEIN C140) |
| LOC647138 | 8.2 X 10^-6^ | 12.86 | 8 | SIMILAR TO KINASE SUPPRESSOR OF RAS-1 (KINASE SUPPRESSOR OF RAS) |
| CNTNAP3 | 7.9 X 10^-6^ | 16.53 | 5 | CONTACTIN ASSOCIATED PROTEIN-LIKE 3 |
| RGPD1 | 7.9 X 10^-6^ | 16.53 | 5 | RAN-BINDING PROTEIN 2-LIKE 1 SHORT ISOFORM |
| UGT3A1 | 7.7 X 10^-6^ | 23.62 | 5 | UDP GLYCOSYLTRANSFERASE 3 FAMILY, POLYPEPTIDE A1 |
| LRRC9 | 7.6 X 10^-6^ | 25.94 | 5 | FLJ46156 PROTEIN |
| AUTS2 | 7.5 X 10^-6^ | 19.08 | 5 | AUTISM SUSCEPTIBILITY CANDIDATE 2 |
| LGR5 | 7.5 X 10^-6^ | 19.08 | 5 | LEUCINE-RICH REPEAT-CONTAINING G PROTEIN-COUPLED RECEPTOR 5 |
| LOC644199 | 7.4 X 10^-6^ | 16.53 | 7 | SIMILAR TO HIGH MOBILITY GROUP PROTEIN 1 (HMG-1) (HIGH MOBILITY GROUP PROTEIN B1) (AMPHOTERIN) (HEPARIN-BINDING PROTEIN P30) |
| LOC727788 | 7.4 X 10^-6^ | 9.8 | 10 |  |
| CNTN1 | 7.3 X 10^-6^ | 26.45 | 5 | CONTACTIN 1 |
| DARS2 | 7.3 X 10^-6^ | 26.45 | 5 | ASPARTYL-TRNA SYNTHETASE 2 (MITOCHONDRIAL) |
| GHR | 7.3 X 10^-6^ | 18.9 | 5 | GROWTH HORMONE RECEPTOR |
| OLFM2 | 7.3 X 10^-6^ | 26.45 | 5 | OLFACTOMEDIN 2 |
| PCDH15 | 7.3 X 10^-6^ | 19.84 | 5 | PROTOCADHERIN 15 |
| ANKRD20B | 7.1 X 10^-6^ | 9.38 | 10 |  |
| KCNB2 | 7.0 X 10^-6^ | 10.17 | 8 | POTASSIUM VOLTAGE-GATED CHANNEL, SHAB-RELATED SUBFAMILY, MEMBER 2 |
| NXPH1 | 6.9 X 10^-6^ | 13.23 | 6 | NEUREXOPHILIN 1 |
| CCDC109A | 6.6 X 10^-6^ | 9.06 | 10 | HYPOTHETICAL PROTEIN FLJ21463 |
| LOC441420 | 6.6 X 10^-6^ | 11.23 | 10 | SIMILAR TO MYOSIN-5B (MYOSIN VB) |
| KCNK9 | 6.5 X 10^-6^ | 14.7 | 6 | POTASSIUM CHANNEL, SUBFAMILY K, MEMBER 9 |
| LOC100128898 | 6.5 X 10^-6^ | 22.96 | 5 |  |
| ARID5B | 6.5 X 10^-6^ | 20.25 | 5 | AT RICH INTERACTIVE DOMAIN 5B (MRF1-LIKE) |
| SNRPN | 6.2 X 10^-6^ | 16.81 | 5 |  |
| SRGAP2P1 | 6.1 X 10^-6^ | 13.78 | 6 | SIMILAR TO SLIT-ROBO RHO GTPASE-ACTIVATING PROTEIN 2 (SRGAP2) (FORMIN-BINDING PROTEIN 2) |
| LOC729458 | 6.1 X 10^-6^ | 22.81 | 5 |  |
| CTNNA3 | 6.0 X 10^-6^ | 23.29 | 5 | CATENIN (CADHERIN-ASSOCIATED PROTEIN), ALPHA 3 |
| LOC100132820 | 5.9 X 10^-6^ | 12.72 | 7 |  |
| CACNA2D3 | 5.8 X 10^-6^ | 16.96 | 6 | CALCIUM CHANNEL, VOLTAGE-DEPENDENT, ALPHA 2/DELTA 3 SUBUNIT |
| NRG1 | 5.6 X 10^-6^ | 19.84 | 5 | NEUREGULIN 1 |
| ANXA7 | 5.6 X 10^-6^ | 23.62 | 5 | ANNEXIN A7 |
| CSMD3 | 5.6 X 10^-6^ | 23.62 | 5 | CUB AND SUSHI MULTIPLE DOMAINS 3 |
| KCTD16 | 5.6 X 10^-6^ | 23.62 | 5 | POTASSIUM CHANNEL TETRAMERISATION DOMAIN CONTAINING 16 |
| OR8K3 | 5.6 X 10^-6^ | 23.62 | 5 | OLFACTORY RECEPTOR, FAMILY 8, SUBFAMILY K, MEMBER 3 |
| SPATA16 | 5.6 X 10^-6^ | 23.62 | 5 | SPERMATOGENESIS ASSOCIATED 16 |
| GRIA1 | 5.3 X 10^-6^ | 8.27 | 9 | GLUTAMATE RECEPTOR, IONOTROPIC, AMPA 1 |
| NLGN1 | 5.1 X 10^-6^ | 28.14 | 5 | NEUROLIGIN 1 |
| SLC35F4 | 5.1 X 10^-6^ | 14.86 | 8 | SOLUTE CARRIER FAMILY 35, MEMBER F4 |
| LOC729852 | 5.0 X 10^-6^ | 22.05 | 5 |  |
| RPA3 | 5.0 X 10^-6^ | 22.05 | 5 | REPLICATION PROTEIN A3, 14KDA |
| MYRIP | 4.7 X 10^-6^ | 18.37 | 5 | MYOSIN VIIA AND RAB INTERACTING PROTEIN |
| RIMS2 | 4.7 X 10^-6^ | 22.65 | 6 | REGULATING SYNAPTIC MEMBRANE EXOCYTOSIS 2 |
| PTPRG | 4.6 X 10^-6^ | 23.62 | 5 | PROTEIN TYROSINE PHOSPHATASE, RECEPTOR TYPE, G |
| PHF20L1 | 4.5 X 10^-6^ | 21.57 | 5 | PHD FINGER PROTEIN 20-LIKE 1 |
| CDH12 | 4.5 X 10^-6^ | 23.29 | 6 | CADHERIN 12, TYPE 2 (N-CADHERIN 2) |
| LOC100132788 | 4.5 X 10^-6^ | 23.29 | 6 |  |
| P4HA1 | 4.5 X 10^-6^ | 22.81 | 5 | PROCOLLAGEN-PROLINE, 2-OXOGLUTARATE 4-DIOXYGENASE (PROLINE 4-HYDROXYLASE), ALPHA POLYPEPTIDE I |
| GTF2IRD1 | 4.3 X 10^-6^ | 16.53 | 5 | GTF2I REPEAT DOMAIN CONTAINING 1 |
| PHACTR1 | 4.3 X 10^-6^ | 22.05 | 5 | KIAA1733 PROTEIN |
| C14orf38 | 4.2 X 10^-6^ | 24.96 | 5 |  |
| FHIT | 4.0 X 10^-6^ | 19.84 | 5 | FRAGILE HISTIDINE TRIAD GENE |
| EPHA6 | 3.9 X 10^-6^ | 22.05 | 5 | EPH RECEPTOR A6 |
| C17orf57 | 3.8 X 10^-6^ | 19.84 | 5 | CHROMOSOME 17 OPEN READING FRAME 57 |
| LOC100132352 | 3.7 X 10^-6^ | 21.33 | 6 |  |
| USH2A | 3.6 X 10^-6^ | 13.78 | 6 | USHER SYNDROME 2A (AUTOSOMAL RECESSIVE, MILD) |
| NTNG1 | 3.6 X 10^-6^ | 18.9 | 6 | NETRIN G1 |
| MDGA2 | 3.5 X 10^-6^ | 22.55 | 5 | MAM DOMAIN CONTAINING 1 |
| C15orf33 | 3.5 X 10^-6^ | 16.53 | 6 | CHROMOSOME 15 OPEN READING FRAME 33 |
| FGF7 | 3.5 X 10^-6^ | 16.53 | 6 | FIBROBLAST GROWTH FACTOR 7 (KERATINOCYTE GROWTH FACTOR) |
| AGBL1 | 3.4 X 10^-6^ | 25.94 | 5 | HYPOTHETICAL PROTEIN FLJ32310 |
| MAGI2 | 3.4 X 10^-6^ | 23.62 | 5 | MEMBRANE ASSOCIATED GUANYLATE KINASE, WW AND PDZ DOMAIN CONTAINING 2 |
| NNT | 3.3 X 10^-6^ | 25.44 | 5 | NICOTINAMIDE NUCLEOTIDE TRANSHYDROGENASE |
| RYR3 | 3.3 X 10^-6^ | 17.1 | 6 | RYANODINE RECEPTOR 3 |
| SLC26A7 | 3.2 X 10^-6^ | 16.53 | 6 | SOLUTE CARRIER FAMILY 26, MEMBER 7 |
| LOC283174 | 2.7 X 10^-6^ | 14.17 | 6 | HYPOTHETICAL PROTEIN LOC283174 |
| SCN1A | 2.7 X 10^-6^ | 17.72 | 5 | SODIUM CHANNEL, VOLTAGE-GATED, TYPE I, ALPHA |
| CNTN4 | 2.6 X 10^-6^ | 24.5 | 6 | CONTACTIN 4 |
| PPP2R2B | 2.6 X 10^-6^ | 22.05 | 6 | PROTEIN PHOSPHATASE 2 (FORMERLY 2A), REGULATORY SUBUNIT B (PR 52), BETA ISOFORM |
| CLSTN2 | 2.6 X 10^-6^ | 18.04 | 5 | CALSYNTENIN 2 |
| LYPLAL1 | 2.6 X 10^-6^ | 20.67 | 5 | LYSOPHOSPHOLIPASE-LIKE 1 |
| CADPS2 | 2.3 X 10^-6^ | 27.56 | 5 | HYPOTHETICAL PROTEIN FLJ20761 |
| KCNH1 | 2.3 X 10^-6^ | 22.81 | 5 | POTASSIUM VOLTAGE-GATED CHANNEL, SUBFAMILY H (EAG-RELATED), MEMBER 1 |
| XYLT1 | 2.1 X 10^-6^ | 19.84 | 5 | XYLOSYLTRANSFERASE I |
| CDKAL1 | 2.1 X 10^-6^ | 33.07 | 5 | CDK5 REGULATORY SUBUNIT ASSOCIATED PROTEIN 1-LIKE 1 |
| NKAIN3 | 2.0 X 10^-6^ | 14.81 | 6 | FAMILY WITH SEQUENCE SIMILARITY 77, MEMBER D |
| BICC1 | 1.9 X 10^-6^ | 22.81 | 5 | BICAUDAL C HOMOLOG 1 (DROSOPHILA) |
| C12orf48 | 1.9 X 10^-6^ | 28.51 | 5 | CHROMOSOME 12 OPEN READING FRAME 48 |
| LDB2 | 1.8 X 10^-6^ | 24.8 | 5 | LIM DOMAIN BINDING 2 |
| FAM149B1 | 1.8 X 10^-6^ | 20.67 | 5 | KIAA0974 |
| LOC728292 | 1.7 X 10^-6^ | 22.05 | 6 |  |
| OPCML | 1.7 X 10^-6^ | 22.05 | 6 | OPIOID BINDING PROTEIN/CELL ADHESION MOLECULE-LIKE |
| FAM55A | 1.7 X 10^-6^ | 17.1 | 6 | FAMILY WITH SEQUENCE SIMILARITY 55, MEMBER A |
| NELL2 | 1.7 X 10^-6^ | 24.8 | 5 | NEL-LIKE 2 (CHICKEN) |
| LOC644459 | 1.7 X 10^-6^ | 25.44 | 5 | HYPOTHETICAL PROTEIN LOC644459 |
| RPS6KC1 | 1.6 X 10^-6^ | 23.62 | 5 | RIBOSOMAL PROTEIN S6 KINASE, 52KDA, POLYPEPTIDE 1 |
| FLJ39080 | 1.5 X 10^-6^ | 26.45 | 5 | HYPOTHETICAL GENE SUPPORTED BY AK096399 |
| COLQ | 1.5 X 10^-6^ | 22.55 | 5 | COLLAGEN-LIKE TAIL SUBUNIT (SINGLE STRAND OF HOMOTRIMER) OF ASYMMETRIC ACETYLCHOLINESTERASE |
| CFTR | 1.4 X 10^-6^ | 23.62 | 5 | CYSTIC FIBROSIS TRANSMEMBRANE CONDUCTANCE REGULATOR, ATP-BINDING CASSETTE (SUB-FAMILY C, MEMBER 7) |
| HHAT | 1.4 X 10^-6^ | 26.45 | 5 | HEDGEHOG ACYLTRANSFERASE |
| SLIT3 | 1.4 X 10^-6^ | 21.2 | 6 | SLIT HOMOLOG 3 (DROSOPHILA) |
| KCNN3 | 1.3 X 10^-6^ | 27.56 | 6 | POTASSIUM INTERMEDIATE/SMALL CONDUCTANCE CALCIUM-ACTIVATED CHANNEL, SUBFAMILY N, MEMBER 3 |
| FER1L6 | 1.3 X 10^-6^ | 33.07 | 5 | C8ORFK23 PROTEIN |
| KCND2 | 1.3 X 10^-6^ | 30.62 | 5 | POTASSIUM VOLTAGE-GATED CHANNEL, SHAL-RELATED SUBFAMILY, MEMBER 2 |
| CRISPLD1 | 1.2 X 10^-6^ | 25.44 | 5 | CYSTEINE-RICH SECRETORY PROTEIN LCCL DOMAIN CONTAINING 1 |
| PARD3B | 1.2 X 10^-6^ | 33.07 | 5 | AMYOTROPHIC LATERAL SCLEROSIS 2 (JUVENILE) CHROMOSOME REGION, CANDIDATE 19 |
| TBC1D5 | 1.2 X 10^-6^ | 2.98 | 82 | TBC1 DOMAIN FAMILY, MEMBER 5 |
| MUC16 | 1.1 X 10^-6^ | 31.2 | 5 | HYPOTHETICAL PROTEIN FLJ14303 |
| ANKRD30A | 1.1 X 10^-6^ | 8.63 | 16 | ANKYRIN REPEAT DOMAIN 30A |
| LOC100132634 | 1.1 X 10^-6^ | 20.67 | 5 |  |
| NELL1 | 1.1 X 10^-6^ | 20.67 | 5 | NEL-LIKE 1 (CHICKEN) |
| PRAMEF13 | 1.1 X 10^-6^ | 24.8 | 5 | PREFERENTIALLY EXPRESSED ANTIGEN IN MELANOMA (PRAME), PSEUDOGENE |
| MAP4K5 | 1.0 X 10^-6^ | 26.45 | 6 | MITOGEN-ACTIVATED PROTEIN KINASE KINASE KINASE KINASE 5 |

The top 286 genes targeted by GBX2 in human PC-3 cells based on ChIP-Seq fragments aligned to the hg18 build of the human genome on the UCSC Human Genome Browser. The top 286 GBX2 target genes are organized by the P values.
