# Supplementary material for: Elongation Factor 1 alpha1 and Genes Associated with Usher Syndromes Are Downstream Targets of GBX2
Source: PLoS One. 2012 Nov 8;7(11):e47366. doi: 10.1371/journal.pone.0047366 (PMC3493575; doi:10.1371/journal.pone.0047366)
Supplement: Table S2 — Primer and oligonucleotide sequences used for identifying and analyzing GBX2 target genes. Data table containing the primer and oligonucleotide sequences used for identifying and analyzing GBX2 target genes. Bold type indicates GBX2 binding sites. (DOC) [file pone.0047366.s003.doc]

**Supplementary Table S2.**  **Primer and oligonucleotide sequences used for identifying and analyzing GBX2 target genes.**

| **Cloning Gbx2 into pCMV-HA** | **Sequences** |
| --- | --- |
| HA-Gbx2-F | 5’ cggggtaccatgagcgca 3’ |
| HA-Gbx2-R | 5’ aatgcggccgctcagggtcgggc 3’ |
| HA-Gbx2dHD-F | 5’ cggggtaccatgagcgca 3’ |
| HA-Gbx2dHD-R | 5’ aatgcggccgcccttatgagcag 3’ |
| **Cloning Gbx2 into pET vector** |  |
| Gbx2-V5-F | 5’ caccatgagcgcagcgttcccg 3’ |
| Gbx2-V5-R | 5’ gggtcgggcctgctccagctgctg 3’ |
| Gbx2d282-V5-F | 5’ caccatgagcgcagcgttcccg 3’ |
| Gbx2d282-V5-R | 5’ caggctggagcagaagccggt 3’ |
| **Gel shift oligos** | **GBX2 binding sequences in bold** |
| PCDH15-F | 5’ttacaatttcatttgtacaggtaa**gtatat**acttttctttcattgtttctttt**taatat**tcccattagtgggaattcaggctatctgtctcttacctatc 3’ |
| PCDH15-R | 5’gataggtaagagacagatagcctgaattcccactaatggga**atatta**aaaagaaacaatgaaagaaaagt**atatac**ttacctgtacaaatgaaattgtaa 3’ |
| PCDH15-F-mutated | 5’ttacaatttcatttgggaattcaggctatctgtctcttacctatc 3’ |
| PCDH15-R-mutated | 5’gataggtaagagacagatagcctgaattcccaaatgaaattgtaa 3’ |
| USH2A-F | 5’acctaatcaaggagaaaagtgcctacaaggt**tataca**cagaccataagagagggatgaatgcatgcatattcttaagacggagagatcactccagtcaag 3’ |
| USH2A-R | 5’cttgactggagtgatctctccgtcttaagaatatgcatgcattcatccctctcttatggtctg**tgtata**accttgtaggcacttttctccttgattaggt 3’ |
| USH2A-F-mutated | 5’acctaatcaaggagaaaagaagacggagagatcactccagtcaag 3’ |
| USH2A-R-mutated | 5’cttgactggagtgatctctccgtcttcttttctccttgattaggt 3’ |
| NOTCH2-F | 5’gagaatcagacaccacaaacattgtatacaaatagaattaagacagaaaa**attaaa**gtcaaggaggtacagagttacttttcaggatgactgaggtttcag 3’ |
| NOTCH2-R | 5’ctgaaacctcagtcatcctgaaaagtaactctgtacctccttgacttta**attttt**ctgtcttaattctatttgtatacaatgtttgtggtgtctgattctc 3’ |
| NOTCH2-F-mutated | 5’gagaatcagacaccacaaacattgtcaggatgactgaggtttcag 3’ |
| NOTCH2-R-mutated | 5’ctgaaacctcagtcatcctgacaatgtttgtggtgtctgattctc 3’ |
| ROBO1-F | 5’ccctacaaaggacatgaactcatgatc**ttttat**ggttgcatagtattccatggtgtatacgtgccgc**attttc**ttaatccagtctatcgttgttggacat 3’ |
| ROBO1-R | 5’atgtccaacaacgatagactggattaa**gaaaat**gcggcacgtatacaccatggaatactatgcaacc**ataaaa**gatcatgagttcatgtcctttgtaggg 3’ |
| ROBO1-F-mutated | 5’ccctacaaaggacatgaactcatccagtctatcgttgttggacat 3’ |
| ROBO1-R-mutated | 5’atgtccaacaacgatagactggatgagttcatgtcctttgtaggg 3’ |
| EEF1A1-F | 5’gtcgtgtactggctccgcctttttcccgagggtgggggagaaccgt**atataa**gtgcagtagtcgccgtgaacgttctttttcgcaacgggtttgccgcca 3’ |
| EEF1A1-R | 5’tggcggcaaacccgttgcgaaaaagaacgttcacggcgactactgcactt**atatac**ggttctcccccaccctcgggaaaaaggcggagccagtacacgac 3’ |
| EEF1A1-F-mutated | 5’ gtcgtgtactggctccgcctctttttcgcaacgggtttgccgcca 3’ |
| EEF1A1-R-mutated | 5’ tggcggcaaacccgttgcgaaaaagaggcggagccagtacacgac 3’ |
| **RT-PCR primers** |  |
| Gbx2-Forward a1 | 5’ gcaacttcgacaaagccgagg 3’ |
| Gbx2-Reverse a1 | 5’ caaattgtcatctgagc 3’ |
| MYO15-rtF1 | 5’ tacatactggatgtggc 3’ |
| MYO15-rtR1 | 5’ cttggacacctgctgta 3’ |
| PCDH15-qPCR F | 5’ cgtggtcaatcaactggatat 3’ |
| PCDH15-qPCR R | 5’ atttctgtcgatggctctgtt 3’ |
| USH2A-qPCR F | 5’ cggccatctaaaggagta 3’ |
| USH2A-qPCR R | 5’ cagatccagtagcagcat 3’ |
| NOTCH2-F1Xho1 | 5’ ccgctcgaggctgctgtcaataat 3’ |
| NOTCH2-R1HindIII | 5’ cccaagcttcgatgtcatggtgcat 3’ |
| **Luciferase oligos** | **GBX2 binding sequences in bold** |
| Eef1a1 F126 Kpn1Xho1 | 5’gtcgtgtactggctccgcctttttcccgagggtgggggagaaccgt**atataa**gtgcagtagtcgccgtgaacgttctttttcgcaacgggtttgccgccagaacacag 3’ |
| Eef1a1 R126 Kpn1Xho1 | 5’ctgtgttctggcggcaaacccgttgcgaaaaagaacgttcacggcgactactgcactt**atatac**ggttctcccccaccctcgggaaaaaggcggagccagtacacgac 3’ |
| Eef1a1 Mutant TATA F1 | 5’gtcgtgtactggctccgcctttttcccgagggtgggggagaaccggcgcgccgtgcagtagtcgccgtgaacgttctttttcgcaacgggtttgccgcca 3’ |
| Eef1a1 Mutant TATA R1 | 5’tggcggcaaacccgttgcgaaaaagaacgttcacggcgactactgcacggcgcgccggttctcccccaccctcgggaaaaaggcggagccagtacacgac 3’ |

Data table containing the primer and oligonucleotide sequences used for identifying and analyzing GBX2 target genes. Bold type indicates GBX2 binding sites.
